# Interaction of IRS2 with PLK1 protects cells from mitotic stress

**DOI:** 10.1038/s41419-026-08706-0

**Published:** 2026-04-08

**Authors:** Ji-Sun Lee, Quyen Thu Bui, Minjeong Jo, Jennifer S. Morgan, Isha Nasa, Arminja N. Kettenbach, Leslie M. Shaw

**Affiliations:** 1 Department of Molecular, Cell & Cancer Biology, University of Massachusetts Chan Medical School, Worcester, MA USA; 2 Department of Pharmacology, School of Medicine, Keimyung University, Daegu, Republic of Korea; 3 Department of Biochemistry and Cell Biology and Dartmouth Cancer Center, Geisel School of Medicine, Dartmouth College, Hanover, NH USA

**Keywords:** Cell division, Growth factor signalling, Chromosome segregation, Kinases, Checkpoints

## Abstract

In this study we identify a role for IRS2 in the protection of cells from mitotic stress through its interaction with PLK1. IRS2 is an adapter protein for the insulin and IGF-1 receptors that mediates their signaling functions. In this capacity, IRS2 is tyrosine phosphorylated to recruit signaling effectors that control cellular outcomes. A role for IRS2 in mitotic regulation has been reported, but the mechanism of IRS2 action in this regulation has not been determined. Here we report that IRS2 interacts with PLK1 in a CDK1-dependent manner. In response to mitotic stress, cells that lack IRS2 or express a PLK1-binding deficient mutant exhibit reduced centrosome separation and a shortened mitotic arrest that leads to reduced tumor cell viability. In contrast, cells expressing an IRS2 mutant that is not tyrosine phosphorylated display normal mitotic function. Together, our findings establish a mechanistic connection between IRS2 and mitotic regulation that is distinct from its function as a signaling adapter protein.

## Introduction

Insulin receptor substrate 2 (IRS2) is a member of the IRS family of adapter proteins that mediate downstream signaling from the insulin (IR) and insulin-like growth factor (IGF1R) receptors in response to engagement of these receptors by their ligands (insulin, IGF1 and IGF2) [1]. Upon recruitment to the activated IR or IGF1R, the IRS proteins are phosphorylated on tyrosine residues by the intrinsic receptor kinases, generating binding sites for effectors to activate downstream signaling and mediate cellular outcomes [2]. The IRS proteins have been shown to play essential roles in the regulation of normal metabolic homeostasis and cell growth by the insulin and IGF signaling (IIS) pathway, with each IRS protein mediating overlapping and distinct functional outputs [3, 4]. In breast cancer, the IRS proteins have also been implicated in divergent functions. IRS1 and IRS2 both support IIS-dependent glucose uptake and cell survival and this can be dependent upon tissue-specific expression of each adapter protein [2]. However, proliferation is induced by IIS only when cells express predominantly IRS1, whereas cells expressing predominantly IRS2 migrate in response to the same signaling input [5, 6]. IRS2 also regulates breast cancer stem cell function in response to IIS through the PI3K-dependent regulation of MYC stability [7]. Recently, IRS2 was identified as a substrate of the anaphase-promoting complex/cyclosome (APC/C)^CDH1^ ubiquitin ligase complex that is activated late in mitosis to allow completion of cell division and return to the G_1_ cell cycle phase [8], revealing the potential involvement of IRS2 in mitosis.

Cell cycle progression is a complex and tightly regulated process which is coordinated by the activation of multiple kinases, regulation of gene expression and targeted protein degradation [9]. Progression is monitored by checkpoints within each cell cycle stage to ensure cellular fitness for cell division. Cells lacking IRS2 were found to have a shortened mitotic phase only when exposed to mitotic stress, indicating a role for IRS2 in the regulation of the mitotic checkpoint, the spindle assembly checkpoint (SAC), which monitors the microtubule engagements by kinetochores to ensure the accurate segregation of chromosomes to maintain genome integrity [10, 11]. Although this study implicates IRS2 in protection from mitotic stress, the mechanism by which IRS2 contributes to this mitotic regulation has not been established.

In the current study, we investigated IRS2 expression and regulation during mitosis in triple negative breast cancer (TNBC) to understand how IRS2 functions in the cellular response to mitotic stress. Our studies reveal a role for IRS2 in PLK1-dependent mitotic regulation that is independent of its function as a signaling adapter for the IIS pathway.

## Results

### IRS2 expression and phosphorylation are regulated during cell cycle progression

**Fig. 1:**
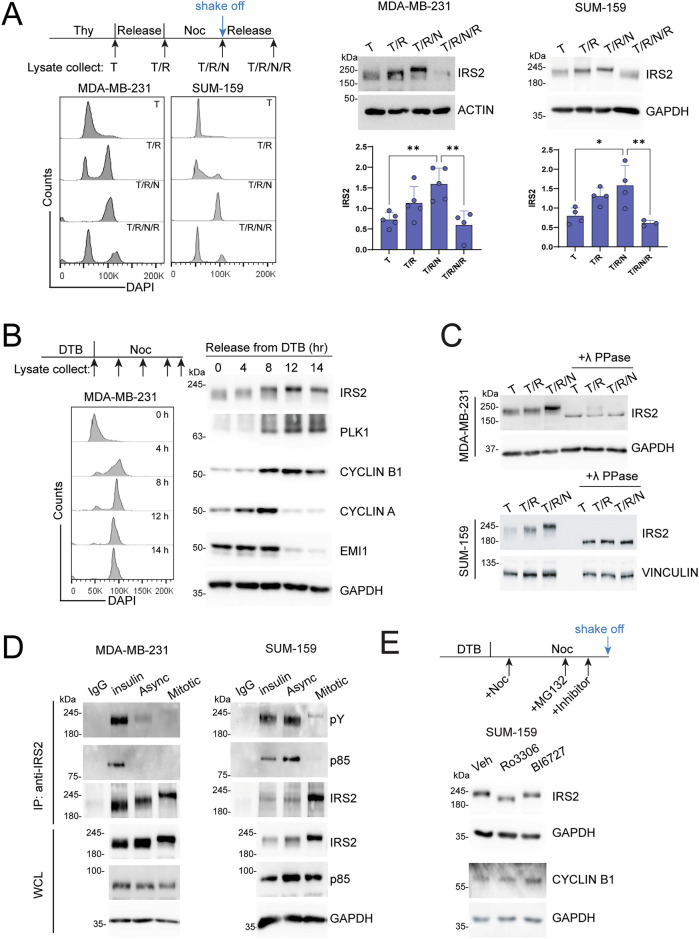
IRS2 expression and phosphorylation are regulated during cell cycle progression. **A** MDA-MB-231 and SUM-159 cells were treated with thymidine (2 mM) for 18 h (T), released for 8 h into normal growth media (T/R), then treated with nocodazole (90 ng/ml) for 16 h. Mitotic cells were collected by shake-off (T/R/N) and then replated and released into growth media for 5 h (T/R/N/R). Cell cycle stages were analyzed by flow cytometry (left) and IRS2 expression was analyzed by immunoblotting (right). The data shown in the graphs represent the mean $\pm \,$SD of three or more independent experiments. *P* values by one-way ANOVA followed by Tukey’s multiple comparison. **B** MDA-MB-231 cells were synchronized by double thymidine block (DTB) and released into growth media in the presence of nocodazole (90 ng/ml). Cell cycle stages were analyzed by flow cytometry (left) and cell extracts were analyzed by immunoblotting for IRS2 and cell cycle proteins. **C** MDA-MB-231 and SUM-159 cells were treated as shown in (**A**) and cell extracts were left untreated or treated with λ phosphatase before analysis by immunoblotting. **D** Cells were serum starved overnight and stimulated with insulin (100 ng/ml) for 10 min (insulin), grown asynchronously in normal growth media (Async), or arrested in mitosis by treatment with nocodazole (90 ng/ml, 16 h) and shake-off (Mitotic). Cell extracts were immunoprecipitated and analyzed by immunoblotting as labeled. **E** SUM-159 cells were synchronized by DTB, released into growth media for 4 h, and subsequently treated with nocodazole (90 ng/ml) for an additional 8 h. 1 h before mitotic cell collection, cells were treated with MG132 (10 uM) and then treated with Ro3306 (10 uM) or BI6727 (100 nM) for the final 30 min before cell collection by shake-off. Cell extracts were analyzed by immunolotting as labeled. All immunoblots are from representative experiments that were replicated at least three times independently.

To study the mechanistic role of IRS2 in mitosis, IRS2 expression during cell cycle progression was assessed in the TNBC cell lines SUM-159 and MDA-MB-231. TNBC models were used because they express elevated levels of IRS2 and they are characterized by a high proliferation rate and chromosome instability, which requires an active mitotic checkpoint to maintain viability [12–14]. Cells were synchronized using a sequential thymidine-nocodazole block and analyzed for cell cycle stage by flow cytometry and IRS2 abundance by immunoblot (Fig. 1A). As previously shown in HeLa cells, IRS2 expression was low in thymidine arrested cells (G_1_/S), increased when released from thymidine for 8 h (G_2_/M), remained elevated after nocodazole treatment to arrest in mitosis (M) and decreased after nocodazole release into normal growth media (return to G_1_). Similar expression changes were observed after palbociclib arrest (G_1_) and release (Fig. S1A). Increased IRS2 protein expression in G_2_/M was also observed using flow cytometry to co-stain for IRS2 and DNA (Fig. S1B). Analysis of mRNA expression under the same treatment conditions revealed that the observed changes in IRS2 protein expression in G_2_/M was not dependent upon transcriptional increases, because IRS2 mRNA expression modestly decreased in G_2_/M arrested cells (Fig. S1C). This result likely reflects a global reduction in mRNA transcription during mitosis that occurs due to chromatin condensation and transcription silencing [15]. Upon exit from mitosis, IRS2 mRNA expression increased, which may reflect the requirement to restore IRS2 protein levels for signaling functions during interphase.

Cell cycle progression is a tightly orchestrated process that is controlled by protein degradation mediated by two families of E3 ubiquitin ligases, the APC/C and the Skp1-Cul1-F-box protein (SCF) complex [16]. APC/C activity is temporally regulated through the combination of two coactivators, CDC20 (required for mitotic progression) and CDH1 (required for G_1_ stabilization). IRS2 was identified as an APC/C^CDH1^-specific substrate that is degraded as cells exit mitosis and re-enter G_1_ [8]. To evaluate more rigorously how IRS2 expression fluctuates during cell cycle progression, cells were arrested at the G_1_/S boundary with a double thymidine block and then released into normal growth medium containing nocodazole to allow cells to progress through S and G_2_ and arrest in M. Cell cycle phases were monitored by flow cytometry and the expression of known cell cycle proteins (Figs. 1B and S1D). Early mitotic inhibitor (EMI1) inhibits both APC/C^CDC20^ and APC/C^CDH1^ during S phase and EMI1 expression is targeted for degradation in early mitosis by the SCF complex [17]. EMI1 expression was high at G_1_/S and decreased after 8 h as cells progressed into G_2_/M. Expression of CYCLIN A gradually increased until 8 h after release and then also decreased at later time points. This pattern reflects prior observations indicating stable CYCLIN A during S/G_2_ and the initiation of degradation immediately after nuclear envelope breakdown facilitated by APC/C^CDC20^ regardless of SAC activation [18–20]. CYCLIN B1 expression increased within 8 h as cells entered into G_2_/M and remained high in cells arrested in mitosis in the presence of nocodazole, reflecting that SAC activation delays its degradation [19]. Expression of the mitotic kinase Polo like kinase 1 (PLK1) also increased within 8 h and remained elevated in nocodazole because of the delayed mitotic exit [21]. The expression pattern of IRS2 was similar to CYCLIN B1 and PLK1, peaking in M and remaining high during delayed mitotic exit.

In addition to cell cycle phase-dependent changes in IRS2 protein expression levels, IRS2 mobility by SDS-PAGE is reduced in mitotic cells (Fig. 1A, B). This IRS2 mobility shift has been previously reported in Hela cells and suggested to be attributed to phosphorylation during mitosis [8]. To directly assess the impact of phosphorylation on the mobility shift of IRS2, protein lysates were treated with λ phosphatase in vitro prior to analysis by SDS-PAGE and immunoblot. After treatment with λ phosphatase, IRS2 mobility was similar and increased across all cell cycle phases, indicating that phosphorylation is responsible for the shift in mobility observed in mitosis (Fig. 1C). In its role as an adapter protein for the IIS pathway, IRS2 is phosphorylated on multiple tyrosine residues by the intrinsic insulin and IGF-1 receptor kinases [2, 22]. To determine if the mobility shift of IRS2 that is observed during mitosis is related to tyrosine phosphorylation of the protein, IRS2 was immunoprecipitated from extracts of insulin stimulated cells, asynchronously growing cells, or mitotic cells. The immune complexes were immunoblotted with antibodies specific for phosphotyrosine or the p85 regulatory subunit of PI3K, which is recruited to IRS2 in a phosphotyrosine-dependent manner. Little to no IRS2 tyrosine phosphorylation or recruitment of p85 was observed in mitotic cells when compared with the levels observed after insulin stimulation (Fig. 1D). IRS2 tyrosine phosphorylation and p85 binding were high in asynchronously growing SUM-159 cells relative to MDA-MB-231 cells, which is attributed to the inclusion of insulin in the normal growth medium of SUM-159 cells. Therefore, increased tyrosine phosphorylation is not the underlying cause of the IRS2 mobility shift in mitosis.

We next assessed the contribution of mitotic regulatory kinases to the mobility shift of IRS2 during mitosis. The serine/threonine kinase PLK1 has been previously reported to directly phosphorylate IRS2 [23]. In addition, inhibition of CDK1, which regulates the transition from G_2_ to M, prevents the mobility shift of IRS2 [8]. However, the inhibition of CDK1 also arrests cells in G_2_ and prevents entrance into mitosis, which could indirectly inhibit the phosphorylation of IRS2. To directly investigate the involvement of PLK1 and CDK1 in the mitosis-dependent phosphorylation of IRS2, cells were synchronized and arrested in mitosis with nocodazole treatment for 12 h before treatment with MG132 to prevent proteasomal degradation. CDK1 and PLK1 inhibitors were added for 1 h before collecting mitotic cells by shake-off and analysis by immunoblotting and flow cytometry (Figs. 1E and S1E, F). Cyclin B1 expression and the percentage of cells in M phase was comparable between the groups across all three conditions confirming that treatment with the inhibitors did not induce premature mitotic exit (Fig. S1F). The CDK1-selective inhibitor Ro3306 reduced the mobility shift of IRS2, whereas the PLK1-specific inhibitor BI6727 did not alter IRS2 mobility significantly, when compared with vehicle control. We conclude from these data that CDK1 plays a major role in inducing the phosphorylation-dependent mobility shift of IRS2 during mitosis.

### IRS2 sustains SAC-dependent mitotic arrest in response to mitotic stress

**Fig. 2:**
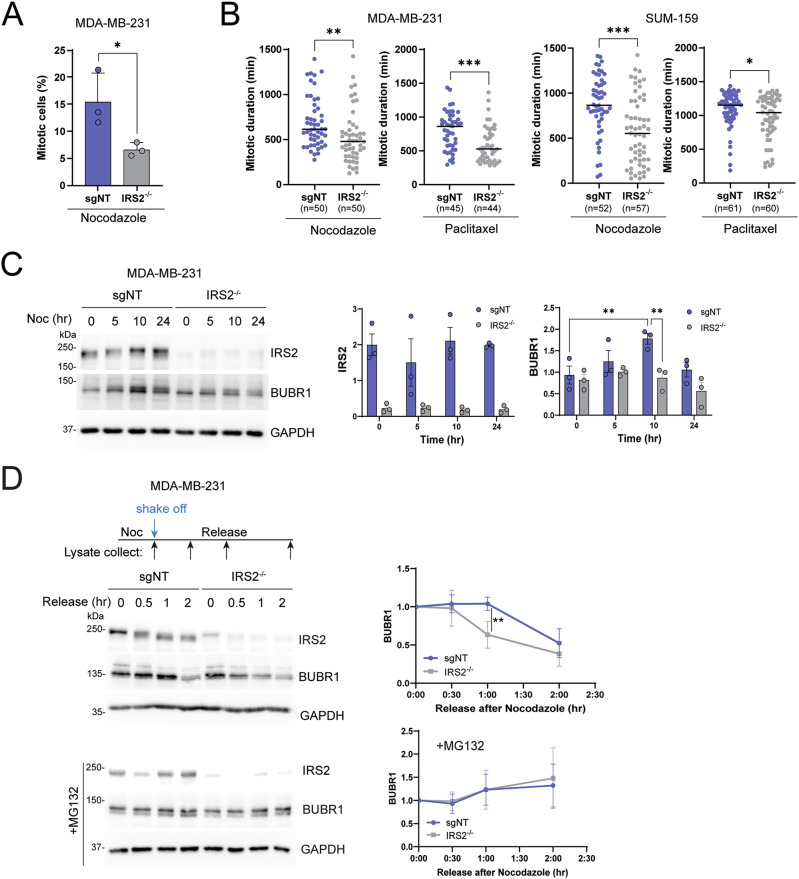
IRS2 sustains SAC-dependent mitotic arrest in response to mitotic stress. **A** H2B-mCherry labeled MDA-MB-231 cells treated with non-targeting guide RNA (*sgNT*) or IRS2 knockout (KO) cells (*IRS2*^*-/-*^) were treated with nocodazole (90 ng/ml). Cells were imaged at 10 h after the treatment, followed by analysis of mitotic cells. The data shown represent the mean $\pm \,$SD of a representative experiment performed three times independently. **B** H2B-mCherry labeled MDA-MB-231 and SUM-159 cells were treated with nocodazole (90 ng/ml) or paclitaxel (100 nM) and imaged every 10 min by widefield time-lapse microscopy for 24 h. Each dot represents the mitotic duration of an individual cell measured as the time from nuclear envelope breakdown to division, slippage, cell death or imaging termination (arrest). The data shown represent the mean $\pm \,$SD of a representative experiment performed three times independently. **C** sgNT or *IRS2*^*-/-*^ MDA-MB-231 cells were treated with nocodazole (90 ng/ml) for the indicated times. Lysates were isolated from both adherent and nonadherent cells and analyzed by immunoblotting as labeled. The data shown in the graphs represent the mean $\pm \,$SD of three independent experiments. **D** *sgNT* or *IRS2*^*-/-*^ MDA-MB-231 cells were treated with nocodazole (90 ng/ml) for 16 h, mitotic cells were collected by shake-off then released into growth media with or without MG132 for the indicated times. Cell extracts were isolated and analyzed by immunoblotting as labeled. The data shown in the graph on the right represent the mean $\pm \,$SD of three independent experiments. *P* values by two-sided *t*-test (**A**,**B**), one-way ANOVA followed by Tukey’s multiple comparison (**C**) or multiple two-sided *t*-test (**D**).

Depletion of IRS2 shortens mitotic duration in cells exposed to mitotic stress, a finding that implicates IRS2 in the regulation of the SAC [8, 24]. This checkpoint is essential for correct chromosome segregation in mitosis by integrating signals from microtubule-unattached kinetochores and delaying anaphase onset until chromosomes are fully attached [10]. We used SUM-159 and MDA-MB-231 cells that were genetically modified by CRISPR/Cas9 gene editing to disrupt IRS2 expression (IRS2^-/-^) to confirm the contribution of IRS2 to the regulation of mitotic arrest in response to mitotic stress (Fig S2A). In asynchronous culture conditions, in the absence of mitotic stress, cell cycle profiles were similar in non-targeting (sgNT) control and IRS2^-/-^ cells (Fig. S2B). Cells were transfected to express mCherry-Histone-H2B to monitor DNA content and exposed to nocodazole for 10 h to induce mitotic stress and activate the SAC [25]. The number of mitotic cells was assessed by imaging of nuclear morphology (Fig S2C). Fewer mitotic cells were present in the IRS2^-/-^ population relative to cells expressing IRS2 (sgNT) after 10 hrs of nocodazole treatment (Fig. 2A), similar to normal RPE cells lacking IRS2 [8].

To investigate further the impact of IRS2 on mitotic duration in response to mitotic stress, time-lapse imaging was used to follow mitotic progression in the presence of nocodazole or the microtubule stabilizing drug paclitaxel. The time from nuclear envelope breakdown (NEB) to anaphase onset was measured in each cell population to monitor mitotic progression. As reported previously, loss of IRS2 did not alter mitotic duration in normal growth conditions (Fig. S2D) [8]. In contrast, in both MDA-MB-231 and SUM-159 cells, IRS2^-/-^ cells displayed shortened mitotic duration under both nocodazole and paclitaxel treatment conditions (Fig. 2B, E). Importantly, the time to mitosis entry did not show a significant difference under any of the treatment conditions (Fig. S2F), providing further evidence that the decreased mitotic duration observed in the absence of IRS2 expression in response to mitotic stress is attributed to early mitotic exit and not delayed mitotic entry [8]. SAC arrest is mediated through the mitotic checkpoint complex (MCC), which consists of four proteins; MAD2, BUBR1, BUB3 and CDC20. BUBR1 is phosphorylated and acetylated during mitosis for stabilization, and its degradation signals SAC silencing [26, 27]. In MDA-MB-231 cells treated with nocodazole, BUBR1 expression increased initially and then decreased with prolonged drug treatment, reflecting SAC activation (10 h) and partial mitotic exit (24 h) (Fig. 2C). In contrast, BUBR1 expression did not accumulate at the 10 h timepoint in IRS2^-/-^ cells, indicating a weak SAC activation. Cells arrested in mitosis by nocodazole treatment were collected and replated into normal growth medium to evaluate their rate of mitotic exit. BUBR1 expression decreased more rapidly in IRS2^-/-^ cells than in sgNT (Fig. 2D), and this loss of expression was rescued by the inclusion of MG132 to inhibit proteosome activity. Taken together, our data support that cells lacking IRS2 elicit a weakened SAC in response to mitotic stress that results in destabilization of the mitotic checkpoint complex and faster mitotic exit.

### IRS2-regulated mitotic arrest prevents mitotic catastrophe

**Fig. 3:**
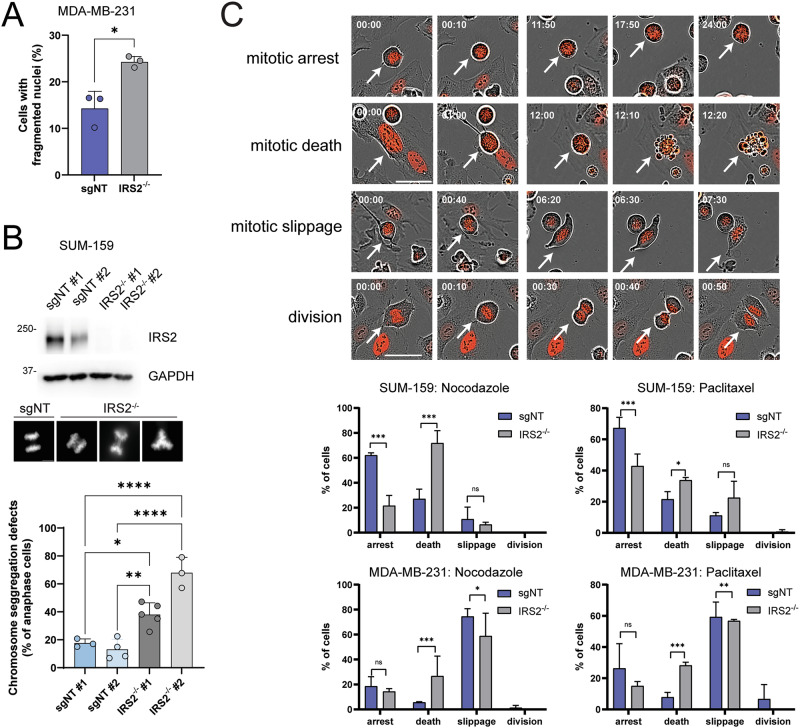
IRS2-regulated mitotic arrest prevents mitotic catastrophe. **A** H2B-mCherry labeled MDA-MB-231 cells treated with non-targeting guide RNA (*sgNT*) or IRS2 knockout (KO) cells (*IRS2*^*-/-*^) were treated with nocodazole (90 ng/ml). Cells were imaged at 30 hours after the treatment, followed by analysis of fragmented nuclei. **B** SUM-159 single cell sgNT and IRS2^-/-^ clones were generated and expression of IRS2 was assessed by immunoblot. Cells were treated with nocodazole (50 ng/ml) for 16 h and then released into growth media for 45 min. Cells were fixed and stained with Hoechst33342 and anaphase cells were imaged. Chromosomal segregation defects were quantified (representative images shown for lagging chromosomes, chromosome bridges and multipolar segregation). The data shown represent the mean $\pm \,$SD of a representative experiment performed three times independently. **C**, **D** Representative images (**C**) and quantification of cell fates (**D**) for each cell line and drug. Scale bar, 50 μm. Time=hr:min. For (**C**), MDA-MB 231 sgNT, nocodazole, *n* = 50; MDA-MB-231 IRS2^-/-^, nocodazole, *n* = 50; MDA-MB 231 sgNT, paclitaxel, *n* = 45; MDA-MB-231 IRS2^-/-^, paclitaxel, *n* = 44; SUM-159 sgNT, nocodazole, *n* = 52; SUM159 IRS2^-/-^, nocodazole, *n* = 57; SUM-159 sgNT, paclitaxel, *n* = 61; SUM159 IRS2^-/-^, paclitaxel, *n* = 60. *P* values by two-sided *t*-test (**A**), one-way ANOVA followed by Tukey’s multiple comparison (**B**) or chi-square test (**D**).

Activation of the SAC arrests cells in prometaphase until all kinetochores have attached properly to the mitotic spindle and chromosomes can be separated with fidelity. If mitotic stress is prolonged and the SAC is functionally deficient, cells may exit mitosis before complete alignment of the chromosomes, leading to chromosomal defects and either aneuploidy or cell death. Indeed, 30 h incubation in nocodazole led to an increase in cells exhibiting nuclear fragmentation, an indication of cell death (Figs. 3A and S2C). To determine if loss of IRS2 expression resulted in abnormal chromosome segregation at mitotic exit, two independent sgNT and IRS2^-/-^ SUM-159 subclones were treated with nocodazole (16 hr) to arrest cells at prometaphase and then released into normal culture media to examine cells as they progress through anaphase. In the absence of IRS2, significantly more cells were observed undergoing abnormal chromosome segregation which included lagging chromosomes, chromosome bridges and multipolar segregation (Fig. 3B). To examine the consequence of IRS2 loss on cellular outcomes in response to prolonged mitotic stress in greater detail, cell fates from the mitotic duration experiment in Fig. 2B were characterized into four categories (Fig. 3C): 1) Arrest: cells remained in mitosis at the endpoint of imaging; 2) Mitotic death: cells underwent apoptosis during mitosis; 3) Slippage: cells exited mitosis without proper cell division; and 4) Division: cells exited mitosis, producing two independent daughter cells. Cell fate outcomes differed between the SUM-159 and MDA-MB-231 sgNT control cells, with the majority of SUM-159 cells arresting in response to drug treatment and the majority of MDA-MB-231 cells exhibiting slippage. However, both cell lines underwent significantly increased cell death when IRS2 expression was absent (Fig. 3D). Collectively these observations support that IRS2 protects cells from cell death in response to mitotic stress by supporting a stable SAC and preventing mitotic catastrophe.

### IRS2 interacts with PLK1 to regulate mitotic progression

As an adapter protein, IRS2 mediates its functions through binding partners. To investigate the mechanism of action of IRS2 in mitosis, we interrogated an open access database, the Biological General Repository for Interaction Datasets (Biogrid)[28], to identify cell cycle regulatory proteins that interact with IRS2 and that could mediate its mitotic functions. Only one protein with a known role in mitotic regulation, PLK1, was identified through this analysis [23, 29, 30]. We also analyzed data from an IRS2 interactome study we had performed using insulin-stimulated SUM-159 cells. Although this interactome study had not been designed to enrich for mitotic cells, we hypothesized that some cells in this asynchronous population were likely in the mitotic state. Analysis of our dataset also revealed a significant interaction of IRS2 with PLK1, and no additional mitotic regulators (Fig S3A and Table S1).

**Fig. 4:**
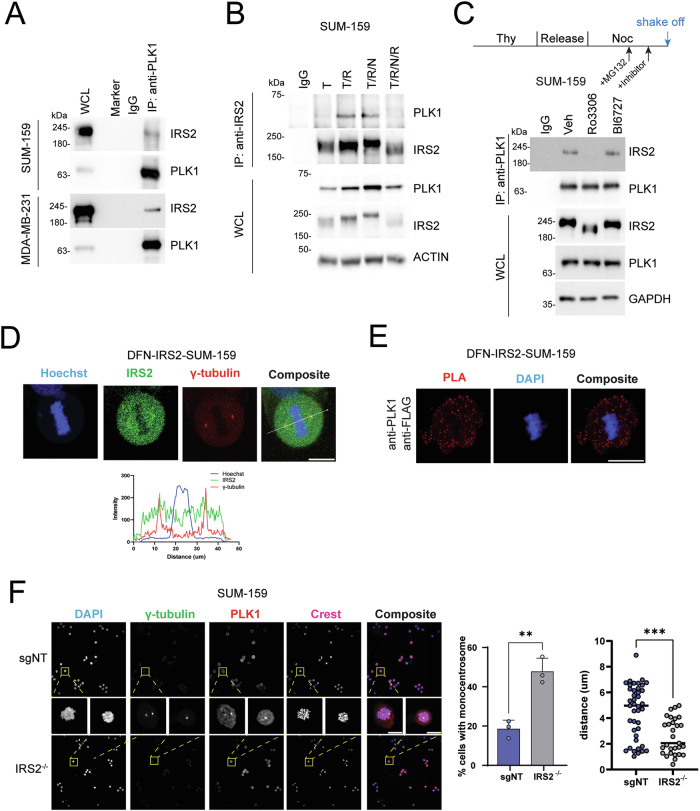
IRS2 interacts with PLK1 during mitosis. **A** SUM-159 and MDA-MB-231 cells were treated with nocodazole (90 ng/ml) for 16 h and cell extracts from mitotic cells were immunoprecipitated with PLK1-specific antibodies. **B** SUM-159 cells were synchronized as shown in Fig. 1A and cell extracts were immunoprecipitated and immunoblotted as labeled. **C** SUM-159 cells were synchronized as shown in the schematic and cell extracts were immunoprecipitated and immunoblotted as labeled. **D** DFN-IRS2 SUM-159 cells were arrested at the G2/M boundary by Ro3306 (10 uM) treatment for 20 h and then released into growth media for 30 min. Cells were imaged for mNeonGreen (IRS2) and γ-tubulin. Scale bar, 20 μm. The plot below represents the line scan analysis of composite image. **E** PLA assay of PLK1 and FLAG in DFN-IRS2 SUM-159 cells. Scale bar, 20 μm (**F**) SUM-159 cells (sgNT and IRS2^-/-^) were arrested in prometaphase by nocodazole treatment (50 ng/ml, 16 h), followed by immunostaining, scale bar, 10 um. Quantification of cells with monocentrosomes determined by γ-tubulin staining (left graph). Quantification of distance between two centrosomes (right graph). sgNT, *n* = 41; IRS2^-/-^, *n* = 29. *P* values by two-sided *t*-test.

PLK1 plays a multifaceted role in mitosis, including the regulation of mitotic entry, centrosome maturation and separation, spindle formation, SAC activation and cytokinesis [31]. To explore the functional significance of the IRS2-PLK1 interaction in the cellular response to mitotic stress, we confirmed that endogenous IRS2 and PLK1 interact in mitotic SUM-159 and MDA-MB-231 cells (Fig. 4A). This interaction is enriched in cells synchronized in the G_2_/M phase of the cell cycle (Fig. 4B) and the binding is dependent upon CDK1 activity, but not PLK1 activity (Fig. 4C). These findings are consistent with a previous PLK1 proteomic interactome study that reported PLK1 binding to IRS2 requires CDK1 activity, but not PLK1 activity [29].

PLK1 expression is enhanced at centrosomes and kinetochores during prophase, prometaphase and metaphase and translocates to the spindle midzone as chromosome segregation occurs in anaphase [31, 32]. IRS2 localization during mitosis is less well understood, with only one report that IRS2 phosphorylated on pS1098 (mouse sequence) localizes to the spindle poles during metaphase [23]. To examine the localization of IRS2 during mitosis, we imaged SUM-159 cells in which mNeonGreen and FLAG tags were inserted into the N-terminus of the endogenous IRS2 locus by CRISPR-Cas9 gene targeting (DFN-IRS2) [33]. As we previously reported, IRS2 is expressed diffusely in the cytoplasm during interphase in these cells (Fig. S3B). Cells were synchronized at G_2_/M with Ro3306 and then released into normal growth medium to enter mitosis. Endogenous IRS2 is diffusely expressed but also shows enrichment at the mitotic spindle and colocalization with γ-tubulin, a marker of centrosomes, in metaphase cells (Fig. 4D). To evaluate the pattern of IRS2 and PLK1 co-localization, a proximity labeling assay (PLA) was performed. A diffuse punctate PLA signal was observed in both prometaphase and metaphase cells when antibodies recognizing FLAG and PLK1 were included together in the assay (Fig. 4E). Individual antibodies showed negligible staining as a negative control for the PLA assay (Fig. S3C).

To determine if IRS2 is required for PLK1 localization during mitosis, sgNT and IRS2^-/-^ SUM-159 cells were arrested in prometaphase with nocodazole and stained for PLK1 and γ-tubulin (Fig. 4F). PLK1 co-localized with γ-tubulin at the spindle pole independently of IRS2 expression, indicating that the interaction between PLK1 and IRS2 is not required for the centrosomal localization of PLK1. However, a 2-fold increase in cells exhibiting monocentrosomes was observed in cells lacking IRS2 expression. Moreover, when two centrosomes were present in IRS2^-/-^ cells, the distance between the centrosomes was significantly reduced. The fluorescence intensity of γ-tubulin staining at monocentrosomes was higher than the staining intensity at double centrosomes, indicating that IRS2 may not be required for centrosome duplication (Fig. S3D). Impaired centrosome separation can lead to defects in bipolar spindle formation and abnormal chromosome segregation, which is consistent with the increased chromosome segregation defects we observed in cells depleted of IRS2 (Fig. 3B).

**Fig. 5:**
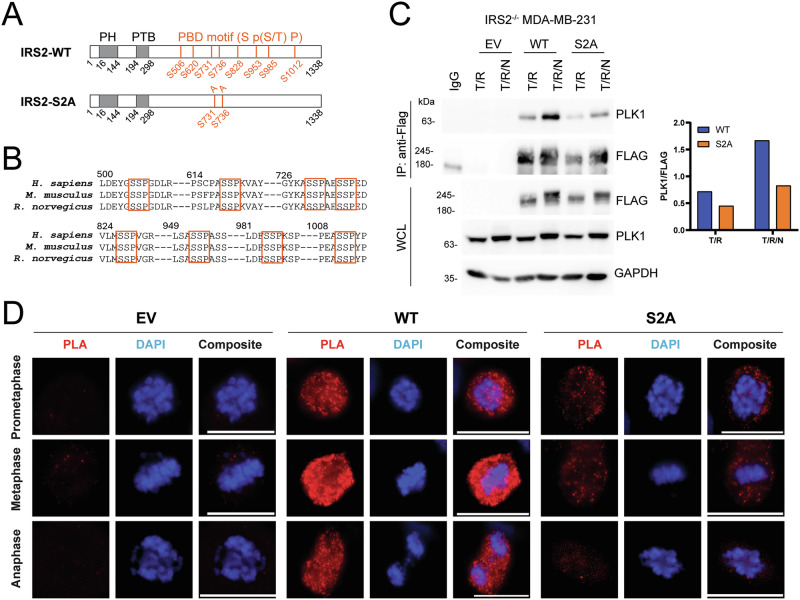
Identification of IRS2 residues that interact with PLK1. **A** Schematic showing IRS2-WT and IRS2-S2A phospho-mutant. PH, pleckstrin homology domain; PTB, phosphotyrosine binding domain; PBD, polo-box domain. **B** IRS2 sequence alignment showing that the PBD motifs (red boxes) are conserved across mammals. **C** *IRS2*^*-/-*^ MDA-MB-231 cells expressing EV, IRS2WT or IRS2-S2A were synchronized by thymidine/release (T/R; G_2_/M) or thymidine/release/nocodazole and shake-off (T/R/N; M). Cell extracts were immunoprecipitated with FLAG-specific antibodies and immunoblotted as labeled. **D** PLA assay of FLAG and PLK1 in SUM-159 IRS2^-/-^ cells restored with EV, IRS2-WT or IRS2-S2A. Cells were treated with nocodazole (50 ng/ml) for 20 h and then released into fresh growth media for 30 min. Scale bar, 20 μm.

We sought to identify the residues of IRS2 that are responsible for the recruitment of PLK1 to investigate the functional significance of the interaction of IRS2 with PLK1 for mitotic regulation. PLK1 consists of two functional domains: the kinase domain (KD) in the N-terminal region and the polo-box domain (PBD) in the C-terminal region. The PBD specifically recognizes PBD motifs (Ser-pSer/pThr-Pro) present in its binding partners [34]. Analysis of the IRS2 sequence revealed eight potential PBD motifs (Fig. 5A), which are conserved across mammals (Fig. 5B). Initially we compared PLK1 binding of IRS2-WT with an IRS2 mutant (IRS2Δ846) that lacks the three C-terminal PBD motifs (S953, S985, S1012). Cells were synchronized with thymidine and nocodazole treatment and the ability of IRS2 to co-immunoprecipitate PLK1 was evaluated. IRS2-WT bound PLK1 in cells in the G_2_/M phase (T/R) and this binding increased in cells enriched in mitosis (T/R/N). The IRS2Δ846 mutant retained the ability to bind to PLK1 at an equivalent level when compared with IRS2-WT (Fig. S4A), indicating that the C-terminal PBD domains are not required for PLK1 binding.

We next examined our LC/MS dataset and determined that of the remaining canonical PBD motifs, only S731 and S736 were phosphorylated. We generated an IRS2 mutant in which both S731 and S736 were mutated to alanine (S731A and S736A; IRS2-S2A) and expressed this mutant in IRS2^-/-^ MDA-MB-231 cells. Binding of PLK1 to the IRS2-S2A mutant was markedly diminished in both G_2_/M and M when compared with IRS2-WT, confirming that S731 and S736 are involved in PLK1 recruitment to IRS2 (Fig. 5C). Given that our LC/MS analysis was performed on cells primarily in interphase, the low residual binding of IRS2-S2A to PLK1 may be due to additional sites that are phosphorylated in a mitosis-specific manner that were not identified in our analysis. The observation that IRS2-S2A still exhibits a mobility shift in mitosis also reflects that additional phosphorylation sites are present. Although we did not observe significant tyrosine phosphorylation of IRS2 in mitotic cells (Fig. 1D), we evaluated the binding of PLK1 to an IRS2 mutant in which five tyrosine residues (Y542, Y653, Y675, Y742 and Y823) located within canonical PI3K binding motifs were substituted with phenylalanine (IRS2-Y5F). Tyrosine phosphorylation of this IRS2 mutant is significantly diminished and it is deficient in its recruitment of PI3K (p85) (Fig. S4B). As a result, cells expressing this mutant exhibit reduced glucose uptake, invade less well and have diminished stemness in response to insulin/IGF signaling [5, 7, 22]. The IRS2-Y5F mutant maintained the ability to interact with PLK1 at WT levels during mitosis (Fig. S4C).

IRS2^-/-^ SUM-159 cells expressing EV or FLAG-tagged IRS2-WT or IRS2-S2A were analyzed for IRS2-PLK1 co-localization using PLA with FLAG and PLK1 antibodies (Fig. 5D). A diffuse, punctate signal was observed in cells restored with IRS2-WT-FLAG expression, similar to the PLA signal detected in DFN-IRS2 cells that express endogenous FLAG-tagged IRS2 (Fig. 4C), although stronger, due to a higher expression of the exogenously expressed IRS2. This PLA signal was evident in prometaphase cells, peaked in metaphase cells and diminished in anaphase cells, reflecting the expression levels of IRS2 during each mitotic phase. In contrast, the PLA signal was markedly reduced in cells expressing IRS2-S2A. Together, these data support the role of S731/S736 in the interaction of PLK1 with IRS2. No PLA signal was detected in IRS2^-/-^ cells (EV).

**Fig. 6:**
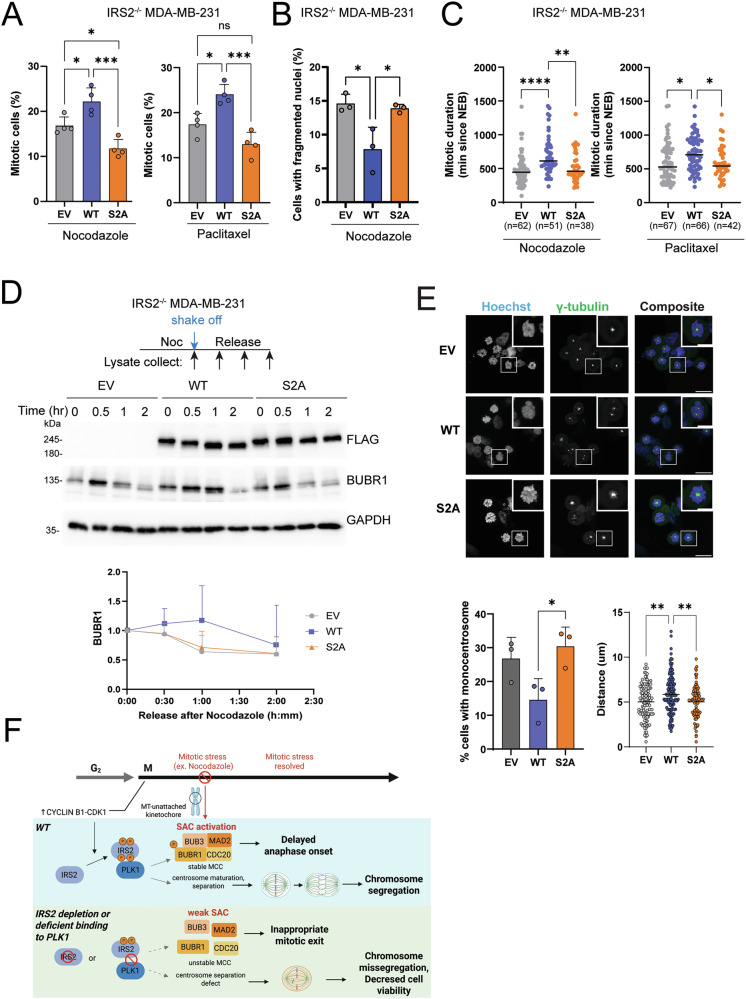
The interaction of IRS2 with PLK1 regulates mitotic progression. **A** *IRS2*^*-/-*^ MDA-MB-231 cells expressing EV, IRS2-WT or IRS2-S2A were treated with nocodazole (90 ng/ml) or paclitaxel (100 nM). Cells were imaged after 10 h of treatment. The data shown represent the mean $\pm \,$SD of a representative experiment performed three times independently. **B** *IRS2*^*-/-*^ MDA-MB-231 cells expressing EV, IRS2-WT or IRS2-S2A were treated with nocodazole (90 ng/ml). Cells were imaged after 30 h of treatment. **C** H2B-mCherry labeled *IRS2*^*-/-*^ MDA-MB-231 cells expressing EV, IRS2-WT or IRS2-S2A were treated with nocodazole (90 ng/ml) or paclitaxel (100 nM). Cells were imaged every 10 min by widefield time-lapse microscopy for 24 h. Each dot represents the mitotic duration of an individual cell measured as the time from nuclear envelope breakdown to division, slippage, cell death or imaging termination (arrest). EV, nocodazole, *n* = 62; WT, nocodazole, *n* = 51; S2A, nocodazole, *n* = 38; EV, paclitaxel, *n* = 67; WT, paclitaxel, *n* = 66; S2A, paclitaxel, *n* = 42. **D** *IRS2*^*-/-*^ MDA-MB-231 cells expressing IRS2-WT or IRS2-S2A were treated with nocodazole (90 ng/ml) for 16 h, mitotic cells were collected by shake-off then released into growth media for the indicated times. Cell extracts were isolated and analyzed by immunoblotting as labeled. The data shown in the graph represents the mean $\pm \,$SD of three independent experiments. **E** *IRS2*^*-/-*^ SUM-159 subclone expressing EV, IRS2WT or IRS2-S2A was arrested in prometaphase by nocodazole treatment (50 ng/ml, 16 h), followed by immunostaining.scale bar, 20 um. Quantification of cells with monocentrosomes determined by γ-tubulin staining (left graph). Distance between two centrosomes (right graph). EV, *n* = 160; WT, *n* = 181; S2A, *n* = 130. **F** Schematic of working model. *P* values by one-way ANOVA followed by Tukey’s multiple comparison.

MDA-MB-231 IRS2^-/-^ cells restored with empty vector (EV), IRS2-WT, IRS2-S2A or IRS2-Y5F were evaluated for their response to mitotic stress. Restoration of IRS2-WT and IRS2-Y5F expression increased the number of mitotic cells relative to EV expressing cells after exposure of cells to either nocodazole or paclitaxel for 10 h (Figs. 6A and S4D). Restoration of IRS2-WT also reduced nuclear fragmentation and lengthened mitotic duration when cells were exposed to prolonged mitotic stress (Fig. 6B, C). In contrast, cells expressing the IRS2-S2A mutant exhibited similar mitotic duration and nuclear fragmentation to EV control cells (Fig. 6A–C). No differences in mitotic entry rate were observed for any of the cell lines (Fig. S5A).

The contribution of IRS2-PLK1 binding to the regulation of mitotic arrest was further demonstrated by the ability of IRS2-WT, but not IRS2-S2A, to sustain expression of BUBR1 and delay mitotic exit after release from nocodazole arrest (Figs. 6D and S5B). Finally, cells expressing EV, IRS2-WT or IRS2-S2A were arrested in prometaphase and stained for Hoechst and γ-tubulin. Restoration of IRS2-WT expression to IRS2^-/-^ cells decreased the number of cells exhibiting monocentrosomes and increased the distance of separation between duplicate centrosomes. The ability of IRS2 to interact with PLK1 was necessary for this rescue as centrosome separation in cells expressing the IRS2-S2A mutant was similar to EV expressing cells (Fig. 6E).

## Discussion

In this study, we identify a role for the adapter protein IRS2 in the cellular response to mitotic stress that is distinct from its well-characterized function as a cytoplasmic signaling adapter. In this latter role, IRS2 is tyrosine phosphorylated by upstream receptor and non-receptor kinases in response to growth factor and cytokine stimuli such as insulin, IGF1 and IL4 to recruit signaling effectors. In contrast, we observe that as cells enter mitosis, IRS2 is phosphorylated on serine/threonine residues, but not tyrosine residues, in a CDK1-dependent manner, and it interacts with the cell cycle regulatory kinase PLK1 (Fig. 6E). The interaction of IRS2 and PLK1 is important for the regulation of centrosome separation to form a bi-polar spindle and maintenance of the SAC in response to mitotic stress. As a result, in cells that lack IRS2 or express a PLK1-binding deficient mutant, centrosome separation is impaired, and mitotic arrest is weakened when cells are exposed to mitotic stress, leading to chromosomal abnormalities and decreased cell viability. Taken together our results support that IRS2 plays a role in protecting cells from mitotic stress that involves its interaction with PLK1 and these mitotic functions of IRS2 do not involve the canonical signaling functions of this adapter protein.

An important question that we addressed in our study relates to how the function of IRS2 in mitosis is connected to its role as a signaling adapter protein during interphase. Previous studies established the importance of IRS2 for the regulation of glucose metabolism, tumor cell migration and invasion and the regulation of MYC and control of breast CSC function by the IIS pathway [5, 7, 22]. In this capacity, IRS2 is tyrosine phosphorylated by the intrinsic insulin and IGF-1 receptor kinases, which generates binding sites for the regulatory subunit of PI3K, as well as other signaling effectors [35, 36]. While the majority of studies on IRS2 function have focused on this role as a cytoplasmic signaling adapter protein during interphase, IRS2 was only recently implicated in regulating the mitotic checkpoint in response to mitotic stress and there was little known about this function [8]. Our current results reveal that the tyrosine phosphorylation of IRS2 and its recruitment of PI3K are not required for the ability of IRS2 to respond to mitotic stress. This conclusion is based upon the lack of tyrosine phosphorylation of IRS2 during mitosis and the ability of an IRS2 mutant that is deficient in tyrosine phosphorylation and PI3K recruitment to retain interactions with PLK1 and to maintain centrosome separation and SAC arrest in response to mitotic stress. These findings indicate that IRS2 has at least two distinct adapter functions that play important roles in different phases of the cell cycle. However, it should be noted that IRS2 is phosphorylated on serine and threonine residues in a feedback manner after its initial tyrosine phosphorylation, which can modify IRS2 signaling functions and regulate protein stability [37]. This regulation has been attributed to downstream signaling kinases such as AKT and mTOR/S6K that feedback to control insulin and IGF-1 signaling to maintain growth and metabolic homeostasis. It is possible that this feedback phosphorylation also contributes to the mitotic functions of IRS2 and serves to connect extracellular signals to the control of cell cycle progression.

PLK1 interacts with binding partners through its PBD, which recognizes SpS/TP motifs [34, 38] Mutation of two serines in IRS2, S731 and S736, diminished the interaction of IRS2 with PLK1 in G_2_/M. Of note, the canonical motif for CDK1 phosphorylation is S/T-P-X-K/R, which does not match the S731 and S736 motif sequences [34, 38]. However, analysis of the IRS2 sequence using the predictive tool Scansite revealed that S731 is located within a low-stringency phosphorylation motif for CDK1 [39]. CDK1 may also function indirectly to activate other mitotic kinases that phosphorylate S731/S736 in IRS2, or CDK1 phosphorylates IRS2 at other sites within IRS2 to prime the phosphorylation of S731 and S736 by other mitotic kinases. Additional CDK1 phosphorylation sites are predicted by Scansite in the IRS2 sequence, as well as phosphorylation sites within motifs that are recognized by the Aurora A/B kinases supporting these alternative mechanisms of phosphorylation.

We performed our IRS2 proteomic study using serum starved and insulin stimulated cells (15 min), a cell population that is predominantly in interphase. Given that the interaction of PLK1 with IRS2 was not completely abolished in the IRS2-S2A mutant, there are likely additional mitosis-specific phosphorylation sites in IRS2 that we did not identify, that either recruit PLK1 directly, or indirectly through a multi-protein complex. It has previously been reported that PLK1 phosphorylates IRS2 on S560 and S1109 (human sequence) and that these phosphorylation events can impact mitotic duration [23]. Although we did not detect a significant mobility shift in IRS2 in the presence of PLK1 inhibitors, it is possible that inhibition of only these two phosphosites does not cause an observable shift on an SDS-PAGE gel. However, upon recruitment to IRS2 in response to CDK1 activation, PLK1-dependent phosphorylation of IRS2 could facilitate other effector interactions that are important for IRS2 function in mitotic regulation. Additional studies of the IRS2 mitotic interactome will be essential to address these open questions regarding mitotic phosphorylation of IRS2.

We identified two essential mitotic functions that are dependent upon the interaction of IRS2 with PLK1. In early mitosis, IRS2 is required for efficient centrosome separation to form a bipolar spindle. Later in mitosis, the IRS2-PLK1 interaction sustains the SAC to ensure that chromosome segregation is delayed until all kinetochores are engaged by the microtubule spindle. Although spindle assembly can occur without centrosomes, or with impaired centrosome separation due to inhibition of PLK1 activity, bi-polar assembly is inefficient, and this can lead to abnormal chromosome segregation and cell death. In some cell types, such as in the epithelial cells of the larval wing disc of Drosophila melanogaster, the loss of centrosomes alone leads to chromosome segregation abnormalities and increased cell death [40]. However, if mitosis is delayed, allowing time for bi-polar spindles to form, some cell types can survive in the absence of centrosomes, such as is observed in larval fly brains [41]. In this instance, a stable SAC is required to delay cell division until an acentrosomal bi-polar spindle can be established [42, 43]. In the absence of a robust SAC, cells with defective centrosome separation undergo increased cell death, as we have observed in cells in which IRS2 is absent or cannot interact with PLK1. In this role, IRS2 serves to support the fidelity of cell division and sustain cell viability.

Chromosomal instability is a common feature in cancer that provides tumor cells with an adaptive advantage and promotes progression [44, 45]. However, the combination of genome instability and high proliferation rates cause mitotic stress, which selects for tumor cells that can adapt and survive under these conditions, because even tumor cells have a loss of viability if aneuploidy exceeds a certain threshold [44, 45]. One mechanism by which tumor cells adapt to prevent mitotic catastrophe is by upregulating SAC genes to ensure that mitosis is not completed until chromosomes are fully attached to the microtubule spindles and can segregate properly [46]. Tumor cells with high SAC function are more resistant to drugs that cause DNA damage and mitotic stress because these cells are more likely to arrest and repair the damage before dividing [47]. The expression of SAC genes is frequently elevated in TNBC, the model used in our study, and high expression is associated with poor overall outcomes [48–50]. TNBC is characterized by high chromosome instability and proliferation rate and requires an active SAC to maintain viability [12–14]. The fact that IRS2 expression is elevated in TNBC, and that it contributes to tumor cell viability in response to mitotic stress, identifies a potential IRS2-specific vulnerability that could be exploited therapeutically in this breast cancer subtype.

## Method and Materials

### Cells, antibodies, and reagents

SUM-159 cells were obtained from BioIVT and authenticated by STR profiling at the University of Arizona Genetics Core. Cells were grown in F12 media (Gibco) containing 5% FBS (Sigma-Aldrich), 5 µg/mL insulin (Sigma-Aldrich) and 1 µg/mL hydrocortisone (Sigma-Aldrich). MDA-MB-231 cells were obtained from the ATCC Cell Biology Collection and were grown in RPMI (Gibco) containing 10% FBS (Sigma). All cells were screened regularly and tested negative for mycoplasma by PCR (Abm).

IRS2 (*IRS2*^*-/-*^) knockout cells were generated by CRISPR/Cas9-mediated gene editing by electroporation of a ribonucleoprotein (RNP) complex of CRISPR-gRNA and Cas9 protein prepared according to manufacturer’s protocol (IDT). Guide RNAs used were as follows: sgIRS2-1, GAG AAC TGC ACG GTG ATC GA; sgIRS2-2, TCG GTA ATG ACC GTG AGC TT. The control non-targeting guide RNA (sgNT) was purchased from IDT.

Human IRS2 was a kind gift from Adrian Lee (University of Pittsburgh, Pittsburgh, PA). cDNA was subcloned into the pCDH-CMV-MCS-EF1-puro lentiviral vector (System Bioscience) with a C-terminal FLAG tag [7]. S731A and S736A mutations were introduced into the pCDH-hIRS2-FFH-EF1 plasmid by site directed mutagenesis using the primers 5’–GAG AGC GCC CCC GAG GAC AGT GGG TAC ATG -3’ and 5’–GGC GGG CGC GCT GGC CTT GTA GCC GCC -3’ according to the manufacturer’s protocol (Q5 Site Directed Mutagenesis Kit; New England Biolabs). The IRS2-Y5F mutant construct was generated by Genewiz and subcloned into the pCDH-CMV-MCS-EF1-puro lentiviral vector (System Bioscience) with a C-terminal FLAG tag. Lentiviruses were generated by co-transfection of IRS2 plasmids and packaging plasmids (pMD2.G and psPAX2) into HEK293FT cells using lipofectamine 3000 according to the manufacturer’s instruction. Cells were infected for 24 h with virus in the presence of 8 ug/ml polybrene (Sigma-Aldrich) and selected with 2 µg/mL puromycin (Gold Biotechnology). pLenti6-H2B-mCherry was a gift from Torsten Wittmann (Addgene plasmid # 89766; http://n2t.net/addgene:89766; RRID:Addgene_89766) [25]. Lentivirus was generated as described above and infected cells were selected with 10 ug/ml blasticidin (Invivogen).

Primary antibodies used in this study include: rabbit anti-IRS2 (Western blotting [WB], 1:1000; Cell Signaling Technology [CST]; # 4502; RRID:AB_2125774), rabbit anti-IRS2 (Immunofluorescence [IF], 1:200; Abcam; # ab134101), Alexa Fluor594 conjugated anti-IRS2 (Flow cytometry, 1:50; Santa Cruz, #sc390761), rabbit anti-PLK1 (WB, 1:1000; CST; #4513), mouse anti-PLK1 (IF, 1:600; Immunoprecipitation [IP], 1:800; Invitrogen; #37-7000), rabbit anti-PLK1 (PLA, 1:500; Invitrogen, #PA5-28023), rabbit anti-γ tubulin (IF, 1:200; Abclonal; #A9657), human anti-CREST (IF, 1:200; Antibodies Incorporated; #15-234), rabbit anti-BUBR1 (WB, 1:2000; Abclonal; #14525), mouse anti-CYCLIN A (WB, 1:2000; Santa Cruz; #271682), rabbit anti-CYCLIN B1 (WB, 1:2000; CST; #4138), mouse anti-EMI1 (WB, 1:1000; Invitrogen; #37-6600), mouse anti-ACTIN (WB, 1:2000; Thermo Fisher Scientific; # MA5-11869; RRID:AB_11004139), mouse anti-GAPDH (WB, 1:2000; Santa Cruz Biotechnology; # sc-32233; RRID:AB_627679), mouse anti-Flag (WB, 1:2000; PLA, 1:500; Sigma-Aldrich; # F3165; RRID:AB_259529), mouse anti-VINCULIN (WB, 1:2000, Santa Cruz Biotechnology; #73614), Alexa Fluor488 conjugated anti-phospho histone H3 (1:200; Biolegend, #650803). Secondary antibodies used in this study include Peroxidase goat anti-Rabbit IgG (Jackson Immunoresearch, #111-035-144), Peroxidase goat anti-Mouse IgG (Jackson immunoResearch, #115-035-146, Goat Anti-Rabbit IgG (H + L) Cross-Adsorbed Secondary Antibody, Alexa Fluor 488 (Invitrogen #A-11008), Goat Anti-Rabbit IgG (H + L) Cross-Adsorbed Secondary Antibody, Alexa Fluor 568 (Invitrogen #A-11011), Goat Anti-Mouse IgG (H + L) Cross-Adsorbed Secondary Antibody, Alexa Fluor 568 (Invitrogen #A-11004), Goat Anti-Human IgG (H + L) Cross-Adsorbed Secondary Antibody, Alexa Fluor 647 (Invitrogen #A-21445).

### Cell cycle synchronization

For single thymidine block, release and nocodazole treatment, cells were treated with thymidine (2 mM, Sigma-Aldrich) for 20 h, released into normal culture media for 8 h, and then treated with nocodazole (90 ng/ml, Sigma-Aldrich) for 16 h. Mitotic cells were collected by shake-off and re-plated in growth media for 5 h. To arrest cells in G_1_, cells were treated with 500 nM (SUM-159 cells) or 150 nM (MDA-MB-231 cells) Palbociclib for 24 h. To arrest cells at the G_1_/S boundary by double thymidine block (DTB), cells were treated with 2 mM thymidine for 20 h, released into normal culture media for 8 h, and then subjected to a second thymidine treatment for 16 h. To arrest cells at the G_2_/M boundary, cells were treated with Ro3306 (10 μM, Selleckchem) for 20 h.

### Immunoprecipitation and immunoblotting

For whole-cell extract immunoblots, cells were solubilized at 4 °C in lysis buffer (20 mM Tris buffer, pH 7.4, 1% Nonidet P-40, 0.137 M NaCl, 10% glycerol) containing phosphatase inhibitors (Roche) and protease inhibitors (Roche). For treatment with λ phosphatase, cells were lysed in buffer without phosphatase inhibitors and cell extracts were treated with λ phosphatase (Santa Cruz) for 1 hr at 30 °C. For immunoprecipitations, cells were extracted in lysis buffer (50 mM Tris buffer, pH 7.4, 1% Triton X-100, 150 mM NaCl) containing phosphatase inhibitors (Roche) and protease inhibitors (Roche). Aliquots of cell extracts containing equivalent amounts of protein were precleared for 1 h with IgG and protein G-Sepharose beads (GE Healthcare) or protein A agarose beads (Thermo) and then incubated for 3 h or overnight at 4 °C with specific antibodies and beads with constant agitation. For IP with FLAG antibody, anti-FLAG M2 affinity gel (Sigma-Aldrich; #A2220) was used. The beads were washed three times in extraction buffer.

Cell extracts containing equivalent amounts of protein or immune complexes were resolved by SDS-PAGE and transferred to nitrocellulose membranes. Membranes were blocked using 5% non-fat milk in 1x TBST (50 mM Tris, pH 7.5, 150 mM NaCl, 0.01% Tween 20). Primary and secondary antibody incubations were carried out in 3% bovine serum albumin (BSA) or 5% non-fat milk in 1X TBST overnight at 4 °C or 1 h at room temperature, respectively. Bands were detected by chemiluminescence using a ChemiDoc XRS+ system (Bio-Rad). For immunoblot quantification, the signals from the protein of interest were normalized with that of the loading control using Image J.

### Quantitative polymerase chain reaction (qPCR)

RNA was extracted using the RNA-easy kit (Qiagen). cDNA was synthesized using an AzuraQuant cDNA synthesis kit (Azura Genomics). qPCR was performed in an Applied Biosystems QuantStudio 6 Flex apparatus using AzuraView GreenFast qPCR Blue Mix (Azura Genomics). The delta –delta Ct method was used to determine relative mRNA expression. Primers used were as follows: h*IRS2*-Fwd 5’-CCA CAT CGT GAA AGA GTG A-3’; h*IRS2*-Rev 5’-CAG AGT CCA CAG ATG TTT CCA A-3’; r18s-Fwd 5’-GTC GCT CGC TCC TCT CCT ACT-3’; r18s-Rev 5’-TCT GAT AAA TGC ACG CAT CCC-3’.

### Flow cytometry

For cell cycle analysis, adherent cells were collected by trypsinization and combined with non-adherent cells from the culture medium. Cells were washed with PBS then fixed in ice-cold 70% ethanol overnight at 4 °C. The fixed cells were washed with PBS twice before treatment with 1 ug/ml DAPI in PBS for 20 min at room temperature. To detect IRS2 or phospho-histone H3, fixed cells were permeabilized by 0.2% tween-20 in PBS treatment for 10 min before incubation with antibodies for 30 min at room temperature. The cells were analyzed by flow cytometry using a ZE5 cell analyzer (Bio-Rad). For PI-based cell cycle profiling, ethanol-fixed cells were incubated with RNase A (50 ug/ml in 0.1% triton X-100/PBS) for 30 min at 37 °C, followed by staining with PI (final concentration 40 ug/ml). The stained cels were analyzed using a BD FACS Canto II, and data were processed with FACSDiva software (BD). All data was processed and analyzed using FlowJo software (v.10.10).

### Mitotic/nuclear fragmentation analysis

Cells were plated in 12 or 24-well plates and treated with nocodazole (90 ng/ml). 10 or 30 h after the treatment, live cells were immediately imaged using an EVOS cell imaging system (Thermo Fisher). Mitotic cells or cells with fragmented nuclei were counted based on nuclear imaging pattern and 200-400 cells per well were analyzed.

### Time-lapse imaging

H2B-mCherry expressing cells were plated in a 96 well plate and incubated overnight. Cells were treated with compounds and imaged immediately afterward. All images were collected using an Incucyte imaging system (Sartorius). Three fields of view were collected per well, and phase contrast and fluorescence images were captured at 10 min intervals for 24 h.

### Immunofluorescence

Cells for immunofluorescence imaging were plated in 8 well chamber slides (Ibidi) or coverslips placed in 24-well plates. Cells were fixed with 4% PFA for 10 min and permeabilized with 0.1% Triton X-100 for 10 min followed by blocking with 1% BSA, 0.3% Triton X-100 in PBS for 30 min at RT. Cells were incubated with primary antibodies (diluted in blocking buffer) for 2–3 h at RT followed by washing with PBS and incubated with AlexaFluor conjugated secondary antibodies in the dark for 1 h with Hoechst 33342 (2 ug/ml). Following washing steps, PBS was added to the chambers and images were collected using a Nikon A1 confocal microscope. Quantification of the signal intensity for the line scan analysis was achieved using Image J. For experiments using DFN-IRS2-SUM159 cells, cells were fixed with 100% methanol at −20 °C for 5 min and processed as described above.

### Proximity ligation assay

2 × 10⁵ cells were seeded onto 12 mm glass coverslips in 12-well plates and incubated overnight. Cells were then treated with 50 ng/mL nocodazole for 20 h, and then released into fresh medium for 30 min. After treatment, cells were fixed with 4% paraformaldehyde for 20 min and permeabilized with 0.2% Triton X-100 for 10 min at room temperature. Protein–protein interactions were subsequently analyzed using the Duolink® In Situ Red Starter Kit (Mouse/Rabbit) (Sigma-Aldrich, DUO92101) according to the manufacturer’s instructions. Following a 1 h block with Duolink Blocking Solution, cells were incubated at room temperature with primary antibodies diluted in Duolink Antibody Diluent. PLA probe incubation, ligation, and amplification were carried out per the Duolink protocol. The cells were then stained with DAPI and coverslips were mounted using anti-fade ProLong Gold. Fluorescence images were captured using an Axio Observer microscope with a ×63 oil immersion objective and controlled by ZEN software (Zeiss).

### LC-MS spectrometry

IRS1^-/-^,IRS2^-/-^ SUM159 cells expressing FLAG-tagged IRS2-WT were serum starved and then stimulated with insulin (500 ng/ml) for 15 min. Cells were extracted in lysis buffer (50 mM Tris, pH 7.4, 150 mM NaCl, 1 mM EDTA, 1% Triton X-100, containing phosphatase inhibitors (Roche) and protease inhibitors (Roche) and cell extracts were clarified by centrifugation (13000 rpm for 15 min at 4 °C). Cell extracts (2 mg) were incubated with agarose-conjugated anti-FLAG antibody (Sigma-Aldrich, 45 ul per IP) for 3 h at 4 °C, then washed three times with lysis buffer. Samples were then incubated with 3x FLAG peptide (0.25 mg/ml, 50 ul per IP, 30 min at 4 °C) to elute the immunoprecipitated proteins. The elution step was performed twice for each sample and then eluents were combined to a single tube.

Proteins from pull-downs were precipitated using 20% tri-chloroacetic acid (TCA), washed twice with acetone (Burdick & Jackson, Muskegon, MI), and digested overnight in 25 mM ammonium bicarbonate with trypsin (Promega) for mass spectrometric analysis. Peptides were analyzed on a Q-Exactive Plus quadrupole equipped with Easy-nLC 1000 (ThermoScientific) and nanospray source (ThermoScientific). Peptides were resuspended in 5% methanol / 1% formic acid and analyzed as previously described [51]. Raw data were searched using COMET (release version 2014.01) in high resolution mode [52] against a target-decoy (reversed) [53] version of the human proteome sequence database (UniProt; 40704 entries of forward and reverse protein sequences) with a precursor mass tolerance of +/- 1 Da and a fragment ion mass tolerance of 0.02 Da, and requiring fully tryptic peptides (K, R; not preceding P) with up to three mis-cleavages. Searches were filtered using orthogonal measures including mass measurement accuracy (+/- 3 ppm), Xcorr for charges from +2 through +4, and dCn targeting a < 1% FDR at the peptide level. Quantification of LC-MS/MS spectra was performed using MassChroQ [54] and the iBAQ method [55].

### Statistical analysis

Statistical analysis was performed using Prism7, Graphpad. Specific statistical tests applied for each dataset are described in the corresponding figure legends. Significance levels were defined as follows: *p < 0.05, **p < 0.01, ***p < 0.001.

## Supplementary information

Supplemental Table

Supplemental Figure Legends

Figure S1

Figure S2

Figure S3

Figure S4

Figure S5

Uncropped western blotting images

checklist

## Data Availability

All data reported in this study will be shared by the lead contact upon request. Any additional information required to reanalyze the data reported in this paper is available from the lead contact upon request. Mass spectrometry data have been deposited to ProteomeXchange: PXD052524 and MSV000094848: https://massive.ucsd.edu/ProteoSAFe/dataset.jsp?task=407565b7b7674af886f18cdd1aaef809. password: 67631.
