# Supplementary material for: Interaction of IRS2 with PLK1 protects cells from mitotic stress
Source: Cell Death Dis. 2026 Apr 8;17(1):495. doi: 10.1038/s41419-026-08706-0 (PMC13187025; doi:10.1038/s41419-026-08706-0)
Supplement: Supplementary file 1 — Supplemental Table [file 41419_2026_8706_MOESM1_ESM.pdf]

**Table S1. Proteins identified and quantified in proteomics (Related to Fig S3A)**

| name | accession | Fold change (stim/unstim) | p-value |
|---|---|---|---|
| EIF3A_HUMAN | Q14152 | 4.43 | 0.0262 |
| RBM6_HUMAN | P78332 | 4.01 | 0.0025 |
| BAG3_HUMAN | O95817 | 3.76 | 0.0110 |
| CKAP5_HUMAN | Q14008 | 3.75 | 0.0056 |
| PRPS1_HUMAN | P60891 | 3.16 | 0.0363 |
| SR140_HUMAN | O15042 | 3.07 | 0.0116 |
| E2F7_HUMAN | Q96AV8 | 3.04 | 0.0045 |
| SF3B1_HUMAN | O75533 | 2.99 | 0.0162 |
| NSRP1_HUMAN | Q9H0G5 | 2.98 | 0.0054 |
| SND1_HUMAN | Q7KZF4 | 2.78 | 0.0271 |
| **PLK1_HUMAN** | **P53350** | **2.75** | **0.0331** |
| HNRPL_HUMAN | P14866 | 2.67 | 0.0302 |
| ANM1_HUMAN | Q99873 | 2.67 | 0.0163 |
| TET2_HUMAN | Q6N021 | 2.51 | 0.0131 |
| POTEI_HUMAN | P0CG38 | 2.31 | 0.0147 |
| ANFY1_HUMAN | Q9P2R3 | 2.29 | 0.0131 |
| CHERP_HUMAN | Q8IWX8 | 2.29 | 0.0485 |
| P85A_HUMAN | P27986 | 2.28 | 0.0042 |
| PABP4_HUMAN | Q13310 | 2.25 | 0.0092 |
| PLEC_HUMAN | Q15149 | 2.22 | 0.0467 |
| NONO_HUMAN | Q15233 | 2.20 | 0.0092 |
| HS74L_HUMAN | O95757 | 2.15 | 0.0118 |
| AKAP2_HUMAN | Q9Y2D5 | 2.14 | 0.0279 |
| SF3B2_HUMAN | Q13435 | 2.12 | 0.0303 |
| POTEE_HUMAN | Q6S8J3 | 2.12 | 0.0221 |
| POTEF_HUMAN | A5A3E0 | 2.12 | 0.0221 |
| POTEJ_HUMAN | P0CG39 | 2.12 | 0.0080 |
| SPTN2_HUMAN | O15020 | 2.09 | 0.0374 |
| ACTC_HUMAN | P68032 | 1.99 | 0.0194 |
| ACTS_HUMAN | P68133 | 1.99 | 0.0194 |
| ACTB_HUMAN | P60709 | 1.97 | 0.0341 |
| ACTG_HUMAN | P63261 | 1.97 | 0.0341 |
| JAK1_HUMAN | P23458 | 1.96 | 0.0196 |
| DHX15_HUMAN | O43143 | 1.96 | 0.0469 |
| HNRPK_HUMAN | P61978 | 1.95 | 0.0308 |
| ACTA_HUMAN | P62736 | 1.94 | 0.0304 |
| ACTH_HUMAN | P63267 | 1.94 | 0.0304 |
| PABP3_HUMAN | Q9H361 | 1.85 | 0.0346 |
| P85B_HUMAN | O00459 | 1.85 | 0.0493 |
| SRC8_HUMAN | Q14247 | 1.58 | 0.0040 |
| F263_HUMAN | Q16875 | 1.30 | 0.0399 |
| TR150_HUMAN | Q9Y2W1 | 1.53 | 0.0122 |
