# Supplementary material for: Interaction of IRS2 with PLK1 protects cells from mitotic stress
Source: Cell Death Dis. 2026 Apr 8;17(1):495. doi: 10.1038/s41419-026-08706-0 (PMC13187025; doi:10.1038/s41419-026-08706-0)
Supplement: Supplementary file 2 — Supplemental Figure Legends [file 41419_2026_8706_MOESM2_ESM.docx]

**Supplementary Figure Legends**

**Figure S1. IRS2 expression and phosphorylation are regulated during cell cycle progression.**  (A) SUM-159 (500 nM) and MDA-MB-231 (150 nM) cells were treated with palbociclib for 24 hrs (P), released for 8 hrs (P/R) then treated with nocodazole (90 ng/ml) for 16 hrs. Mitotic cells were collected by shake-off (P/R/N) then replated and released into growth media for 5 hrs (P/R/N/R). Cell extracts were analyzed by immunoblotting for IRS2 and cell cycle proteins. (B) *IRS2^-/-^* MDA-MB-231 cells expressing empty vector (EV) or IRS2-WT were treated as shown in Fig 1A. T/R or T/R/N cells were collected and fixed, and IRS2 expression was analyzed by flow cytometry. (C) MDA-MB-231 cells were treated with thymidine (2 mM) for 18 hrs (T), released for 8 hrs (T/R) then treated with nocodazole (90 ng/ml) for 16 hrs. Mitotic cells were collected by shake-off (T/R/N) then replated and released into growth media for 5 hrs (T/R/N/R). The data shown represent the mean ± S.D. of *IRS2* mRNA expression from three independent experiments. *P* values by one-way ANOVA followed by Tukey’s multiple comparison. (D) SUM-159 cells were synchronized by double thymidine block and released into growth media in the presence of nocodazole (90 ng/ml). Cell extracts were analyzed by immunoblotting for IRS2 and cell cycle proteins. (E) MDA-MB-231 cells were treated as shown in Figure 1E, and cell extracts were analyzed by immunoblotting for IRS2. (F) SUM-159 cells were treated as shown in Figure 1E. Cell cycle stages were analyzed by PI staining and flow cytometry.

**Figure S2. IRS2 sustains SAC-dependent mitotic arrest in response to mitotic stress.**  (A) MDA-MB-231 or SUM-159 cells treated with non-targeting guide RNA (*sgNT*) or IRS2 knockout (KO) cells (*IRS2^-/-^*) were analyzed for IRS2 expression by immunoblotting. (B) Asynchronously grown cells were stained with DAPI and analyzed by flow cytometry for cell cycle profiles. (C) Representative images of H2B-mCherry labeled *IRS2^-/-^* MDA-MB-231 cells treated with nocodazole (90 ng/ml, 10 hrs) from Figures 2A, 3A, 6A and 6B showing interphase, mitotic and fragmented nuclei. (D) *sgNT* or *IRS2^-/-^* cells growing asynchronously in complete growth media were imaged every 10 minutes by widefield time-lapse microscopy for 24 hrs. Each dot represents the mitotic duration of an individual cell measured as the time from nuclear envelope breakdown to anaphase onset or completion of division. *sgNT* SUM-159, n=65; *IRS2^-/-^* SUM-159, n=61; *sgNT* MDA-MB-231, n=64; *IRS2^-/-^* MDA-MB-231, n=60. (E) Representative images of SUM-159 cells expressing H2B-mCherry from Figure 2B. Scale bar, 50 μm. Time=hr:min. (F) Quantification of time to mitosis-entry, with time from drug treatment to nuclear envelope breakdown plotted as cumulative frequency (from Figure 2B). *P* values by two-sided t-test (D) or log-rank (Mantel-Cox) test (F).

**Figure S3. IRS2 interacts with PLK1 during mitosis.** (A) *Top,* schematic of experimental process for mass spectrometry analysis. IRS1^-/-^/IRS2^-/-^ SUM159 cells expressing FLAG-tagged IRS2-WT were serum starved and then stimulated with insulin (500 ng/ml) for 15 min. Cell extracts were immunoprecipitated with anti-FLAG antibodies followed by LC/MS analysis. *Bottom,* Volcano plot comparing proteomes of unstimulated vs stimulated IRS2-WT expressing cells. (B) Interphase DFN-IRS2 SUM-159 cells were imaged for IRS2. Scale bar, 20 μm. (C) PLA assay of FLAG and PLK1 in SUM-159 IRS2^-/-^ cells restored with IRS2-WT. Cells were treated with nocodazole (50 ng/ml) for 20 hrs and then released into fresh growth media for 30 min. Scale bar, 40 μm. (D) Quantification of γ-tubulin immunostaining intensity in IRS2^-/-^ cells from the immunofluorescence images in Figure 3G. *P* values by two-sided t-test.

**Figure S4. Identification of IRS2 residues that interact with PLK1.** (A) IRS1,2^-/-^ SUM-159 cells expressing, EV, IRS2WT, IRS2Δ846 were synchronized as shown in Figure 1A. Cell extracts were immunoprecipitated with FLAG-specific antibodies and immunoblotted as labeled. (B) Cell extracts from cells stimulated with insulin (100 ng/ml, 10 min, left) or growing asynchronously in complete growth media (right) were immunoprecipitated with FLAG or IRS2-specific antibodies and immunoblotted as labeled. (C) IRS2^-/-^ SUM-159 cells were treated with nocodazole (90 ng/ml) for 16 hrs and cell extracts from mitotic cells were immunoprecipitated with FLAG-specific antibodies. (D) *IRS2^-/-^* MDA-MB-231 cells expressing EV, IRS2-WT or IRS2-Y5F were treated with nocodazole (90 ng/ml) or paclitaxel (100 nM). Cells were imaged after 10 hours of treatment. The data shown represent the mean $\pm$ SD of a representative experiment performed three times independently. *P* values by one-way ANOVA followed by Tukey’s multiple comparison.

**Figure S5. The interaction of IRS2 with PLK1 regulates mitotic progression.** (A) H2B-mCherry labeled *IRS2^-/-^* MDA-MB-231 cells expressing EV, IRS2-WT or IRS2-S2A were treated with nocodazole (90 ng/ml) or paclitaxel (100 nM). Cells were imaged every 10 minutes by widefield time-lapse microscopy for 24 hrs. Time to mitosis-entry was quantified, with time from drug treatment to nuclear envelope breakdown plotted as cumulative frequency (From Figure 4B). (B) Densitometric quantification of FLAG (IRS2) expression at time 0 from Figure 6D. *P* values by log-rank (Mantel-Cox) test (A) or two-sided t-test (B).
