# Supplementary figures and images for: Interaction of IRS2 with PLK1 protects cells from mitotic stress
Source: Cell Death Dis. 2026 Apr 8;17(1):495. doi: 10.1038/s41419-026-08706-0 (PMC13187025; doi:10.1038/s41419-026-08706-0)

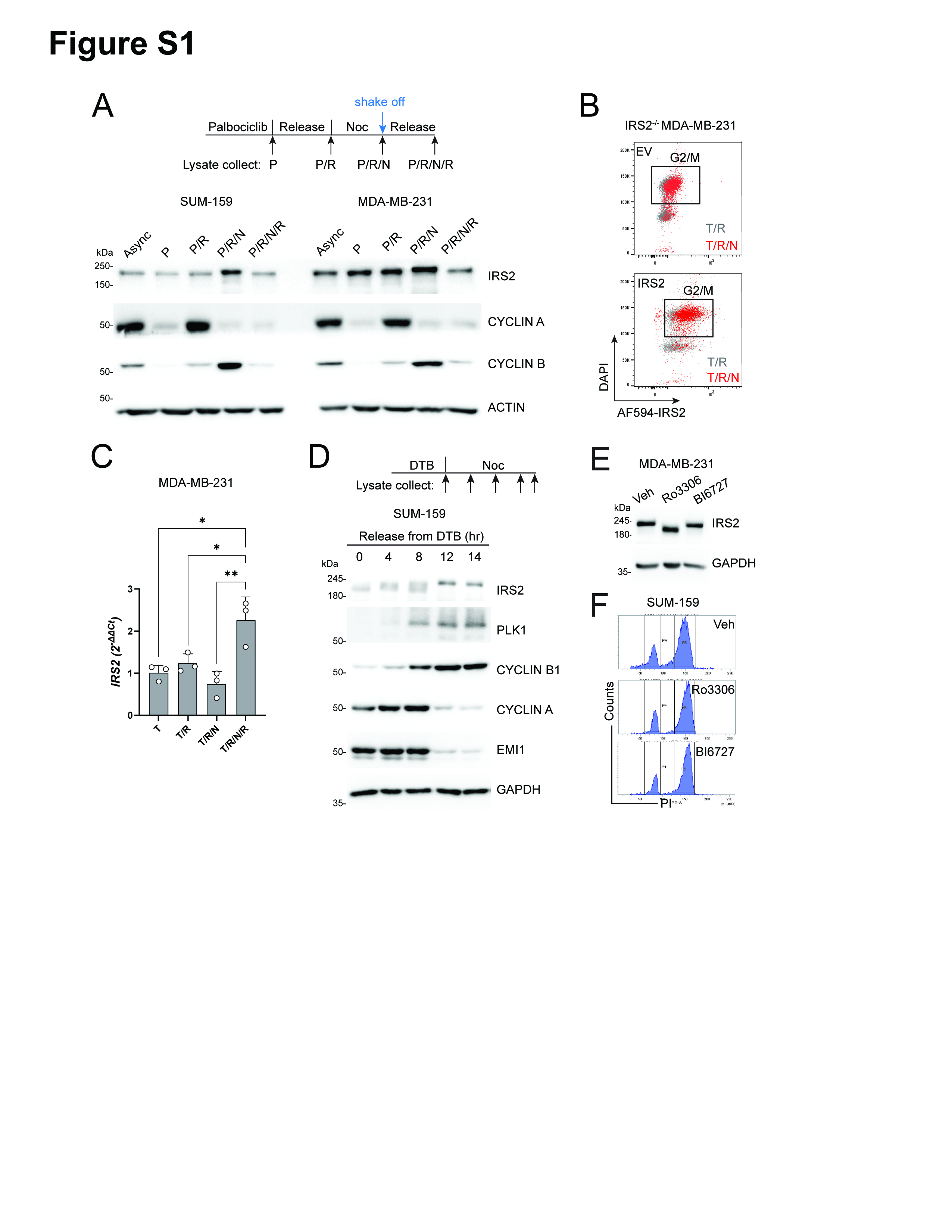

Supplement: Supplementary file 3 — Figure S1 [file 41419_2026_8706_MOESM3_ESM.tif]

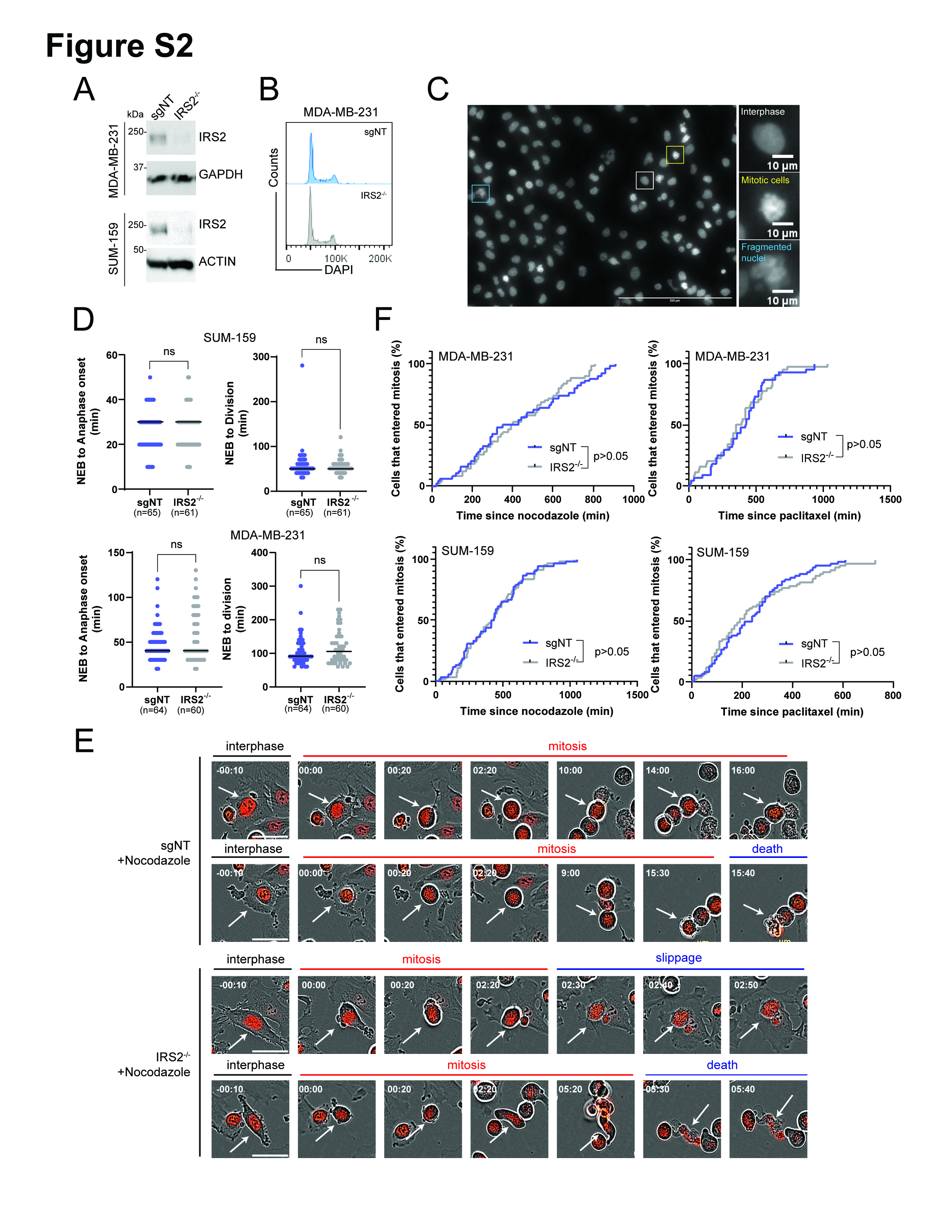

Supplement: Supplementary file 4 — Figure S2 [file 41419_2026_8706_MOESM4_ESM.tif]

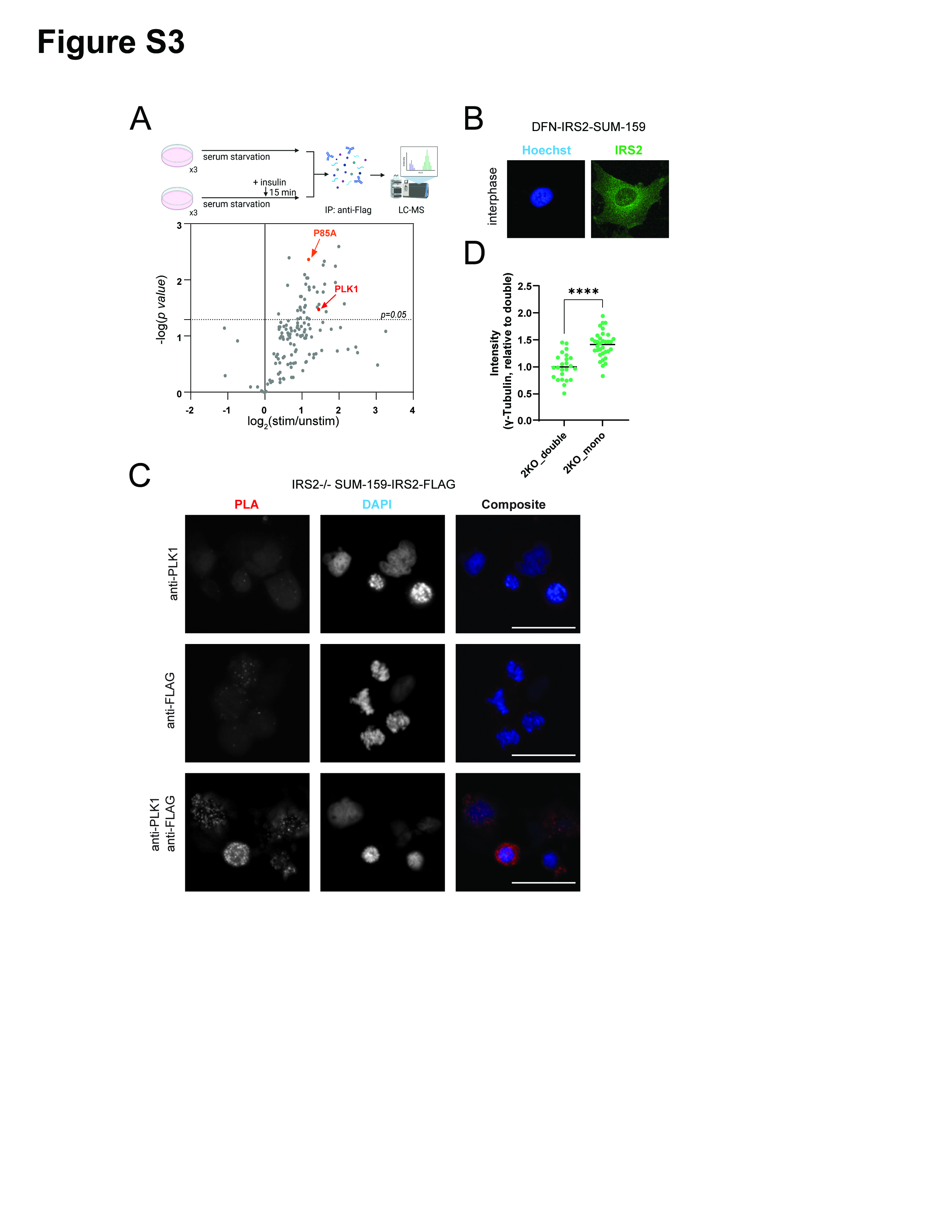

Supplement: Supplementary file 5 — Figure S3 [file 41419_2026_8706_MOESM5_ESM.tif]

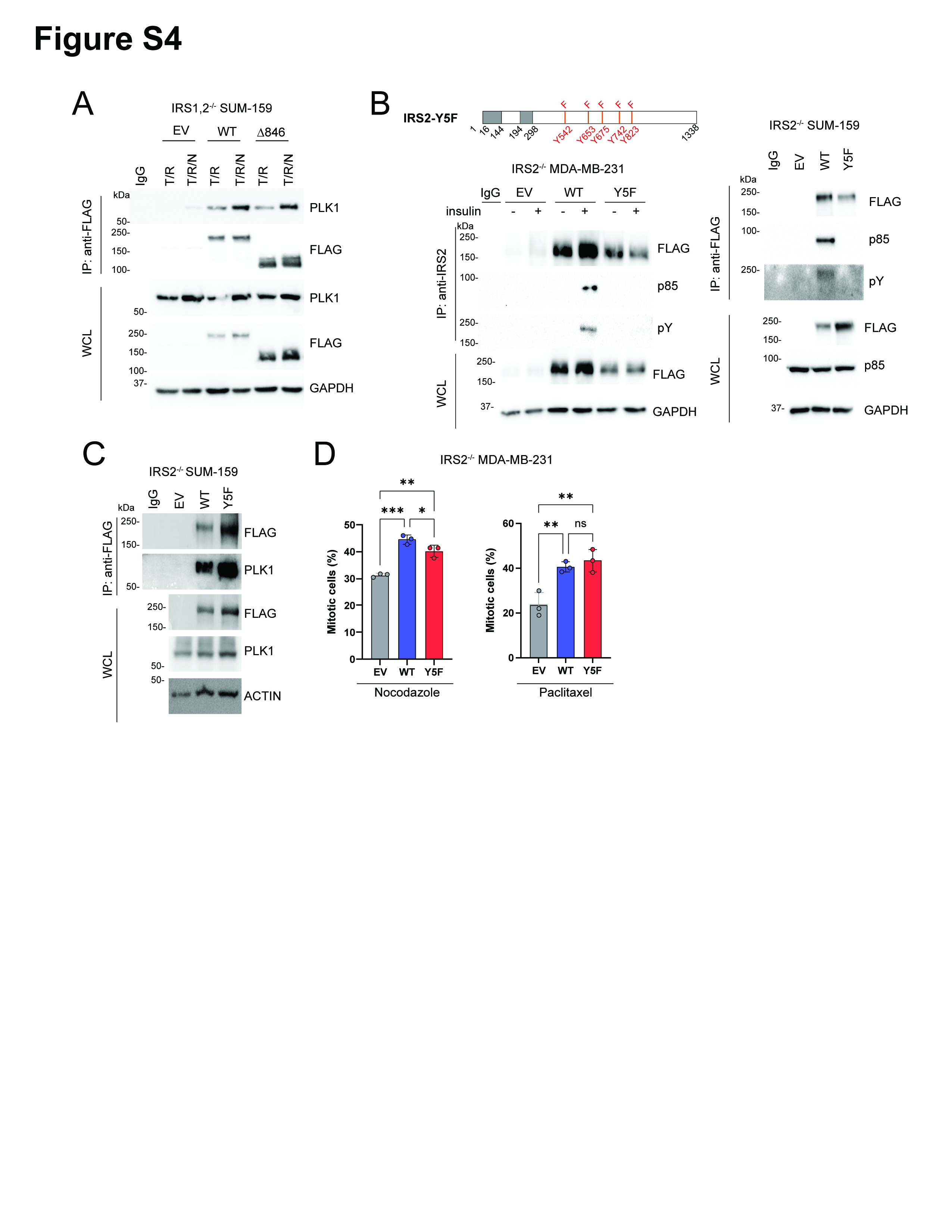

Supplement: Supplementary file 6 — Figure S4 [file 41419_2026_8706_MOESM6_ESM.tif]

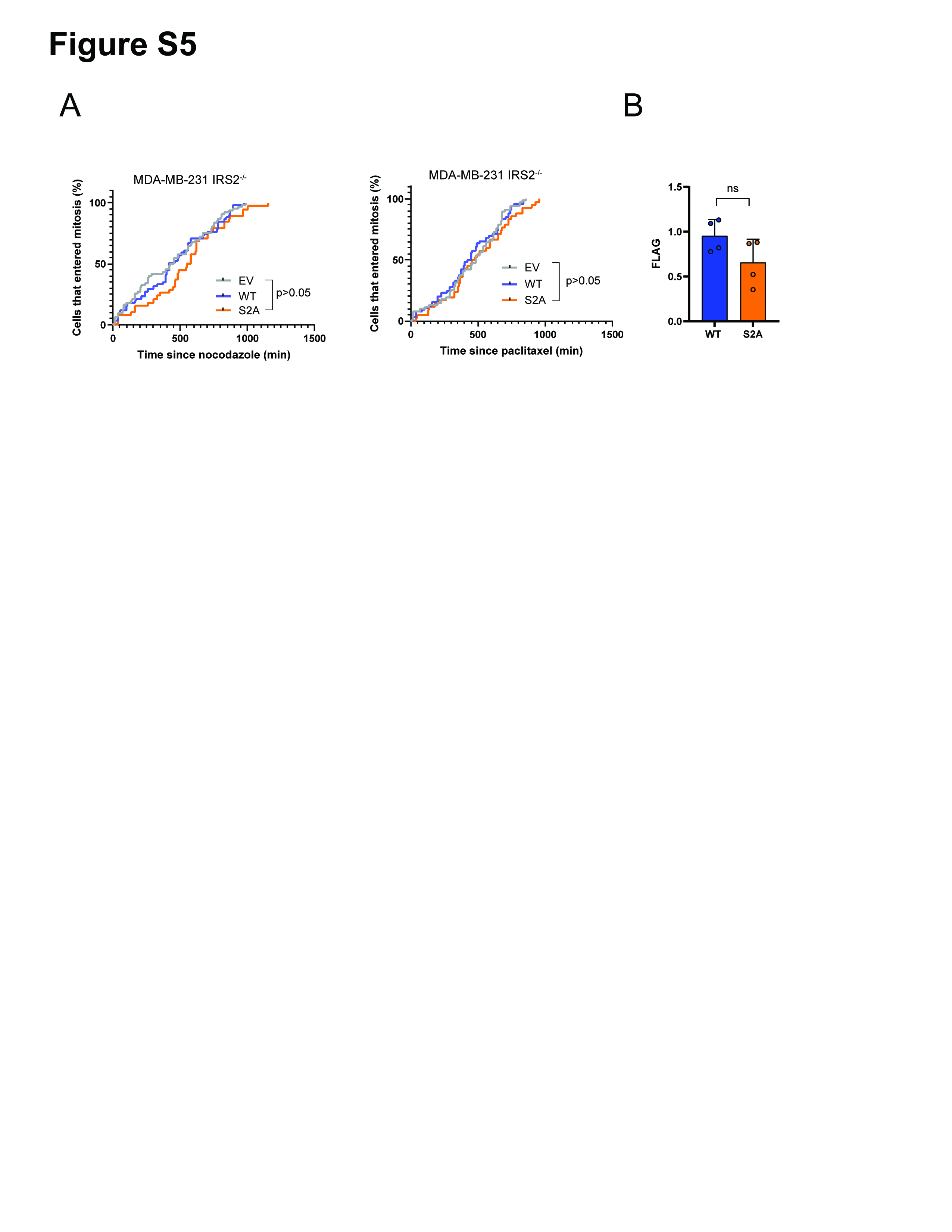

Supplement: Supplementary file 7 — Figure S5 [file 41419_2026_8706_MOESM7_ESM.tif]

Fig. 1A

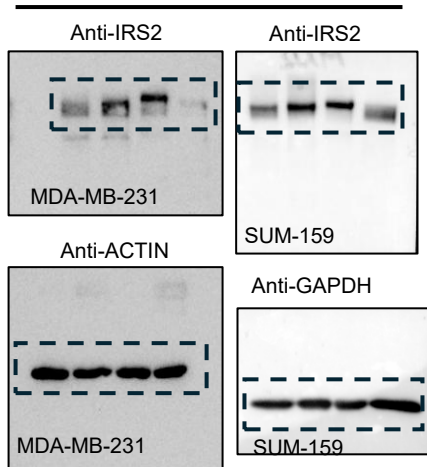

Fig. 1B

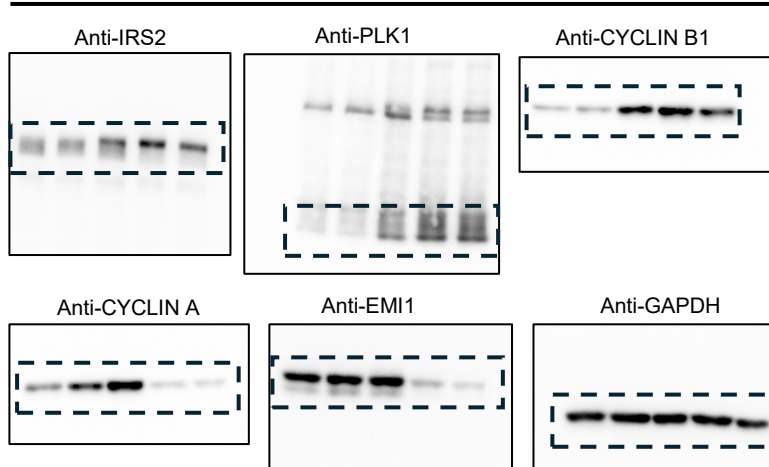

Fig. 1C

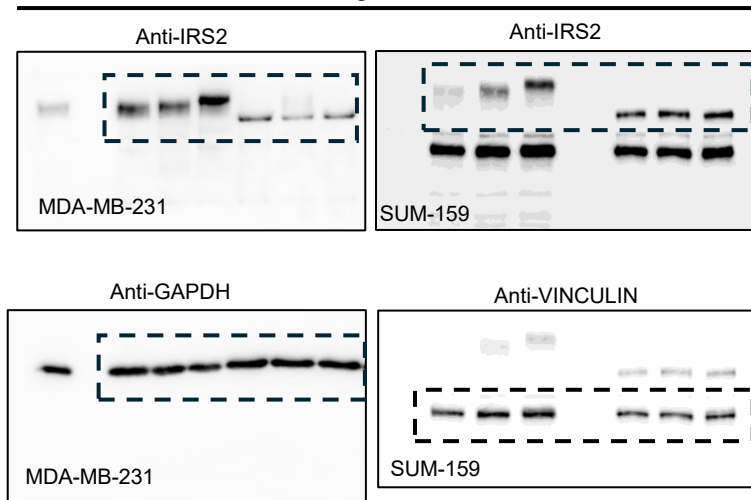

Fig. 1E

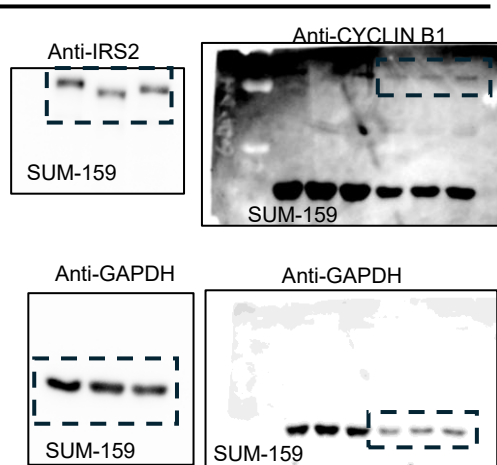

Fig. 1D

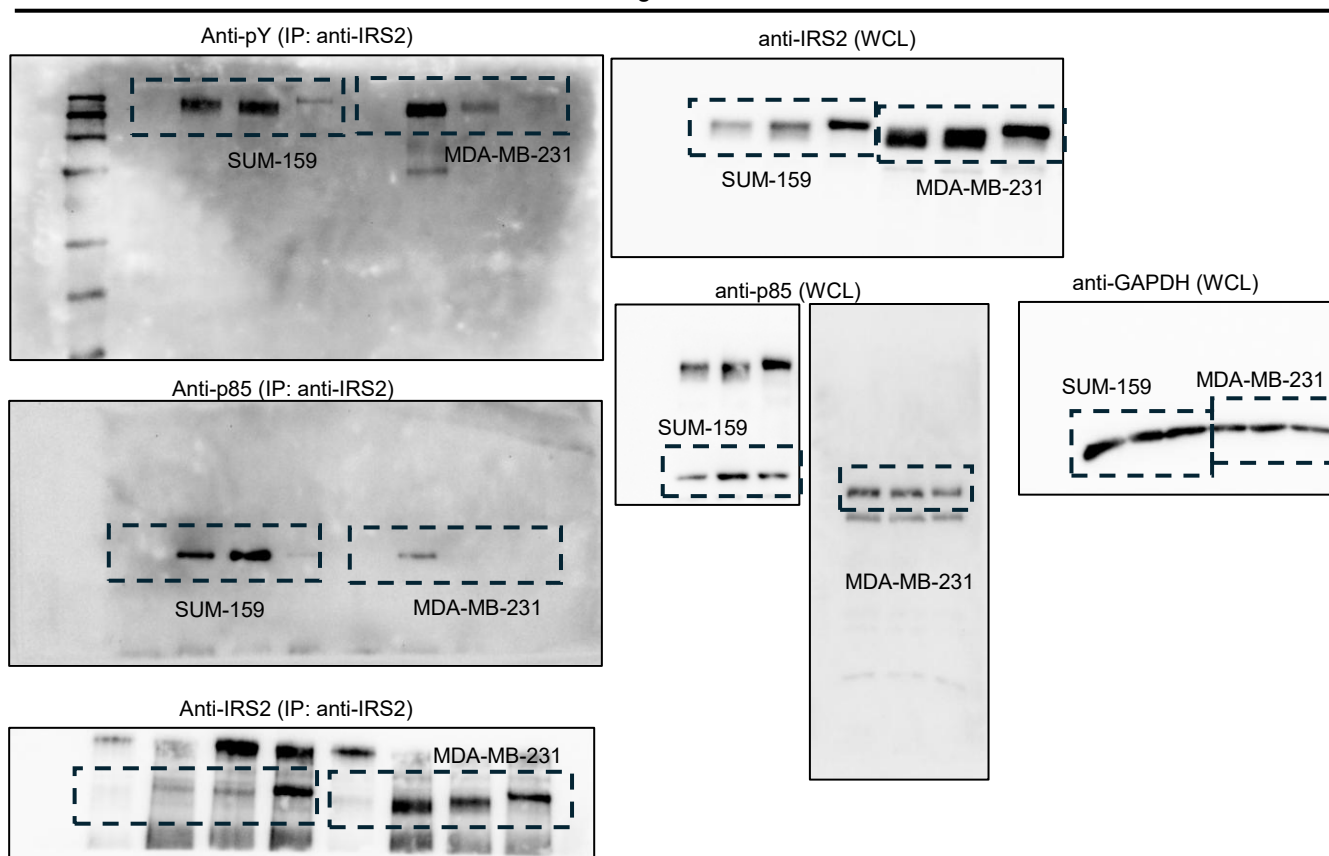

Fig. 2C

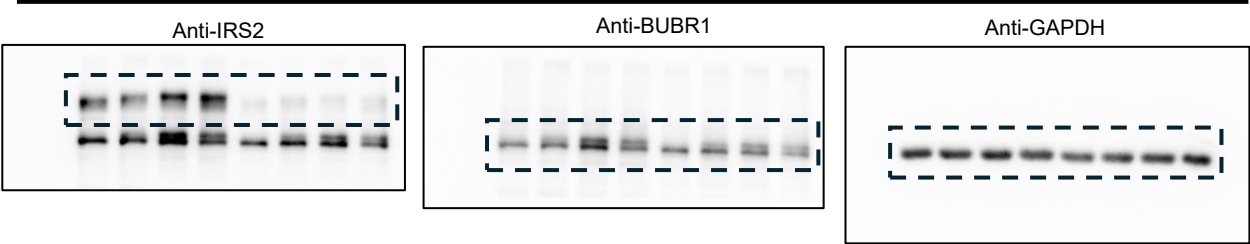

Fig. 2D

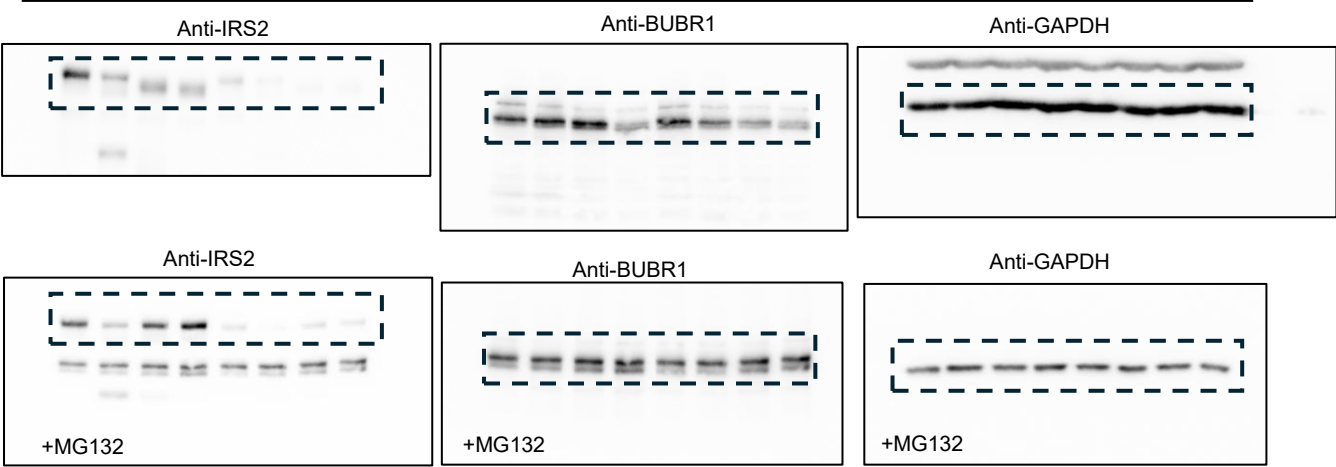

Fig. 3B

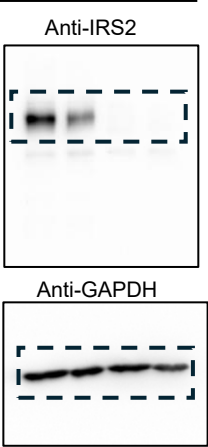

Fig. 4A

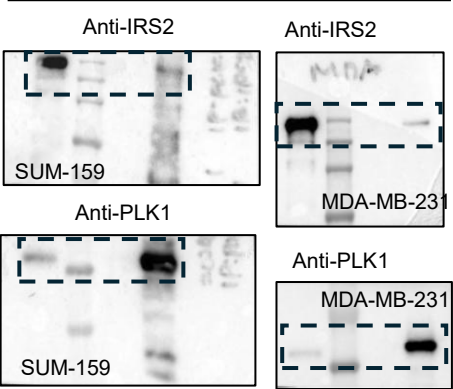

Fig. 4B

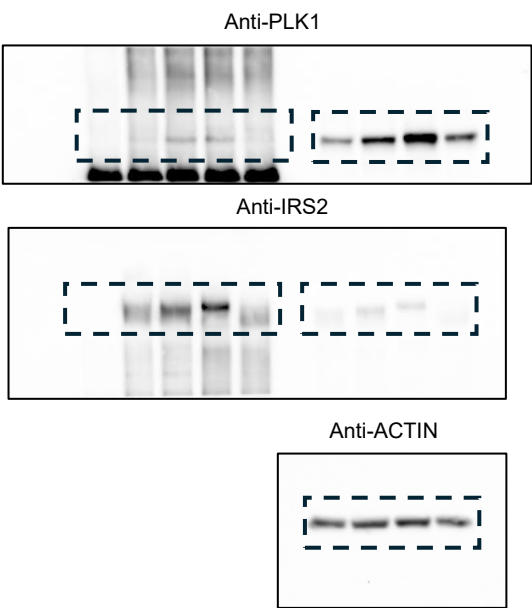

Fig. 4C

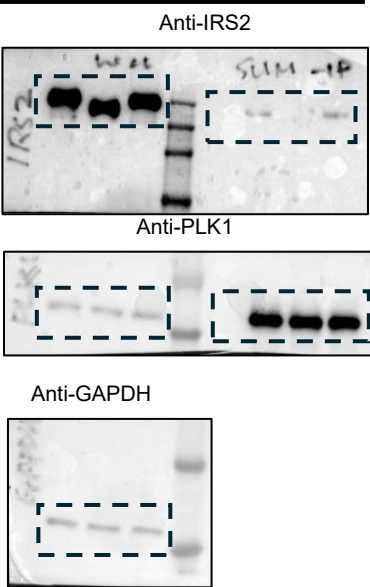

Fig. 5C

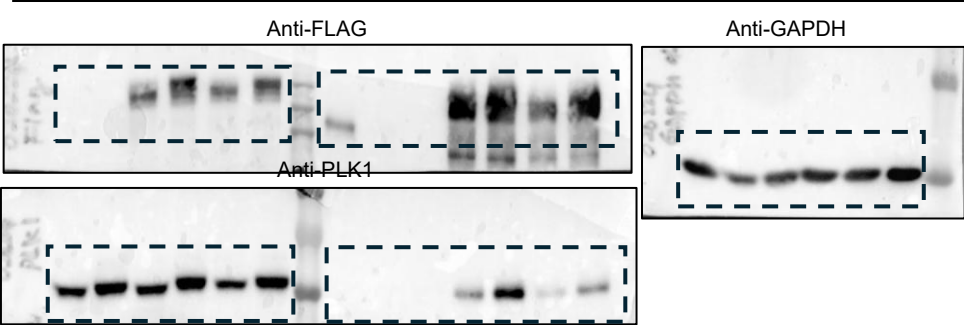

Fig. 6C

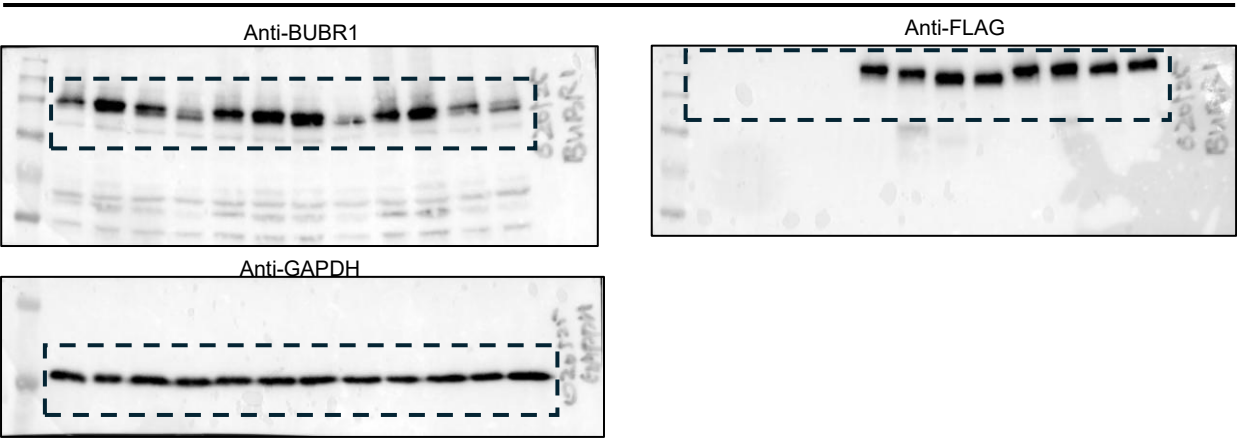

Fig. S1A

Fig. S1D

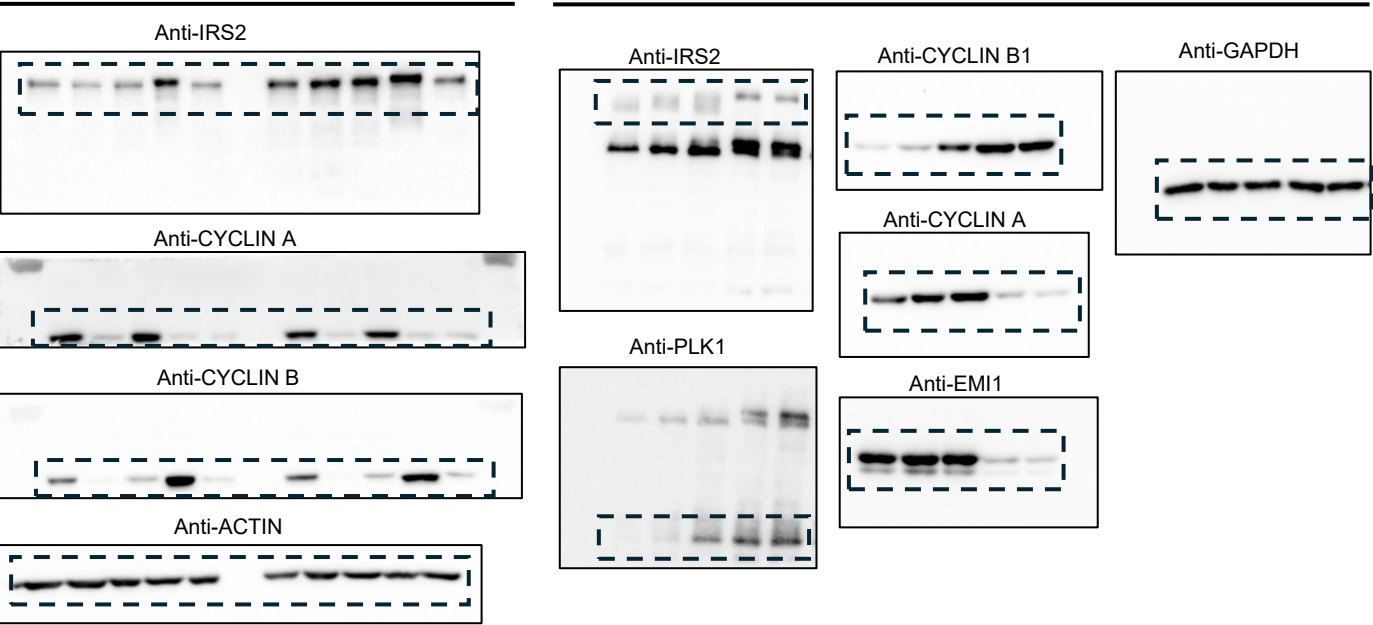

Fig. S2A

Fig. S4A

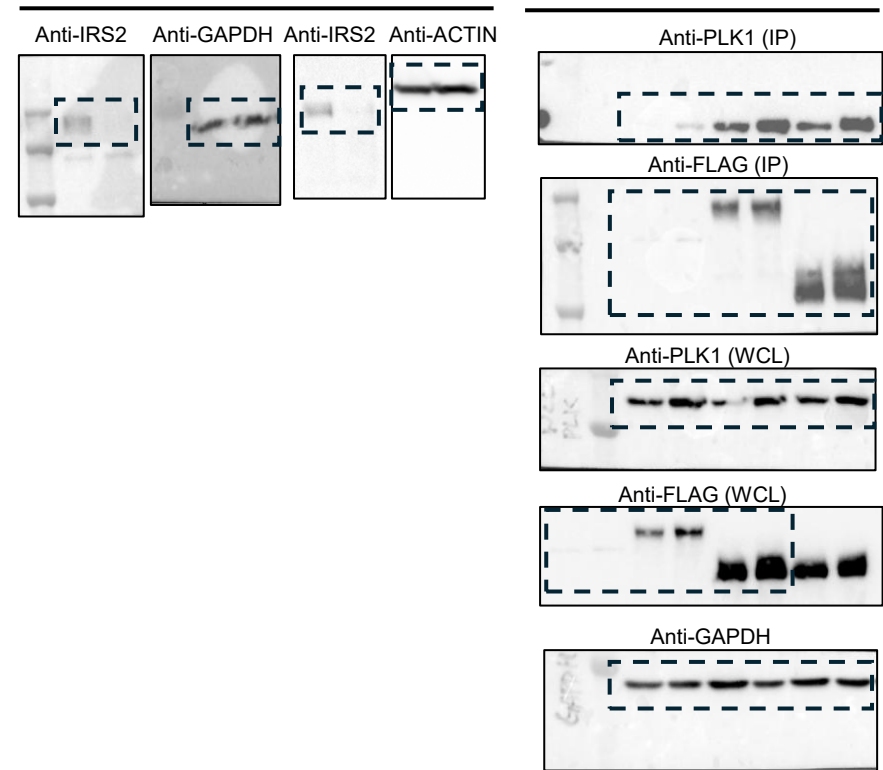

Supplement: Supplementary file 8 — Uncropped western blotting images [file 41419_2026_8706_MOESM8_ESM.pdf]
